# Neurogenin 3 Expressing Cells in the Human Exocrine Pancreas Have the Capacity for Endocrine Cell Fate

**DOI:** 10.1371/journal.pone.0133862

**Published:** 2015-08-19

**Authors:** Danielle L. Gomez, Marci O’Driscoll, Timothy P. Sheets, Ralph H. Hruban, Jose Oberholzer, James J. McGarrigle, Michael J. Shamblott

**Affiliations:** 1 Children’s Research Institute, Department of Pediatrics, University of South Florida Morsani College of Medicine, St. Petersburg, FL, United States of America; 2 Department of Gynecology and Obstetrics, John Hopkins University, Baltimore, MD, United States of America; 3 Departments of Pathology and Oncology, The Sol Goldman Pancreatic Cancer Research Center, Johns Hopkins University School of Medicine, Baltimore, MD, United States of America; 4 Department of Surgery, University of Illinois at Chicago, Chicago, IL, United States of America; 5 Department of Bioengineering, University of Illinois at Chicago, Chicago, IL, United States of America; University of Szeged, HUNGARY

## Abstract

Neurogenin 3 (NGN3) is necessary and sufficient for endocrine differentiation during pancreatic development and is expressed by a population of progenitor cells that give rise exclusively to hormone-secreting cells within islets. NGN3 protein can be detected in the adult rodent pancreas only following certain types of injury, when it is transiently expressed by exocrine cells undergoing reprogramming to an endocrine cell fate. Here, NGN3 protein can be detected in 2% of acinar and duct cells in living biopsies of histologically normal adult human pancreata and 10% in cadaveric biopsies of organ donor pancreata. The percentage and total number of NGN3+ cells increase during culture without evidence of proliferation or selective cell death. Isolation of highly purified and viable NGN3+ cell populations can be achieved based on coexpression of the cell surface glycoprotein CD133. Transcriptome and targeted expression analyses of isolated CD133+ / NGN3+ cells indicate that they are distinct from surrounding exocrine tissue with respect to expression phenotype and Notch signaling activity, but retain high level mRNA expression of genes indicative of acinar and duct cell function. NGN3+ cells have an mRNA expression profile that resembles that of mouse early endocrine progenitor cells. During *in vitro* differentiation, NGN3+ cells express genes in a pattern characteristic of endocrine development and result in cells that resemble beta cells on the basis of coexpression of insulin C-peptide, chromogranin A and pancreatic and duodenal homeobox 1. NGN3 expression in the adult human exocrine pancreas marks a dedifferentiating cell population with the capacity to take on an endocrine cell fate. These cells represent a potential source for the treatment of diabetes either through *ex vivo* manipulation, or *in vivo* by targeting mechanisms controlling their population size and endocrine cell fate commitment.

## Introduction

Endocrine hormones secreted by pancreatic islets maintain glucose homeostasis throughout life. During rodent development, islets arise from progenitor cells expressing the transcription factor neurogenin 3 (NGN3), which is necessary and sufficient for endocrine specification [1–5] and is similarly expressed during human pancreas development [6–8]. The role of NGN3 in the adult pancreas is unclear. NGN3 cannot be routinely detected in the rodent pancreas but knockout has a negative impact on adult islet function [9]. Upregulation by dedifferentiating beta cells [10, 11] suggests NGN3 may mark loss of mature function or represent a less committed progenitor cell state.

Although the cell lineage, timing and mechanisms of islet development have been established, the processes maintaining islet mass throughout life remain in question. Estimates of human beta cell longevity suggest islet formation is completed early in life and that beta cells persist with limited proliferation compared to rodents [12, 13]. Murine lineage-tracing studies suggest that preexisting beta cells [14–17], not exocrine cells [18, 19], are the predominant source of regenerating beta cells under normal circumstances and following certain types of experimental pancreatic injury [14–19]. However, other cells within islets [20–22] and exocrine cells [23–35] are capable of generating insulin expressing cells and islet-like structures following injury or *in vitro* manipulation. A role for NGN3 in the formation of islets in the adult pancreas (beta cell and islet neogenesis) is also difficult to establish. NGN3 expression following injury is insufficient to drive transdifferentiation of duct cells into an endocrine cell fate [36]. However, beta cell neogenesis has been demonstrated from exocrine cells that transiently express NGN3 following adenoviral expression [35], partial duct ligation [27, 28], 90% pancreatectomy [37, 38], *in vivo* delivery of EGF and CNTF [39] or LIF [40], *in vivo* knockdown of E3 ligase Fbw7 [41], expression of STAT3 and MAPK [42] and *in vivo* expression of PDX1, MAFA and NGN3 [43]. Although these results do not demonstrate exocrine to endocrine reprogramming or transdifferentiation under normal *in vivo* circumstances, they establish that exocrine cells have the capacity to take on an endocrine cell fate and strongly suggest a role for NGN3 in this process.

Here, we describe the expression of NGN3 protein in biopsies of histologically normal adult human exocrine pancreas. The phenotype and *in vitro* differentiation of isolated NGN3+ cells suggest they are dedifferentiating exocrine cells with the capacity to take on endocrine fate.

## Results

### NGN3 Is Expressed by Acinar and Duct Cells in the Adult Human Pancreas

**Fig 1:**
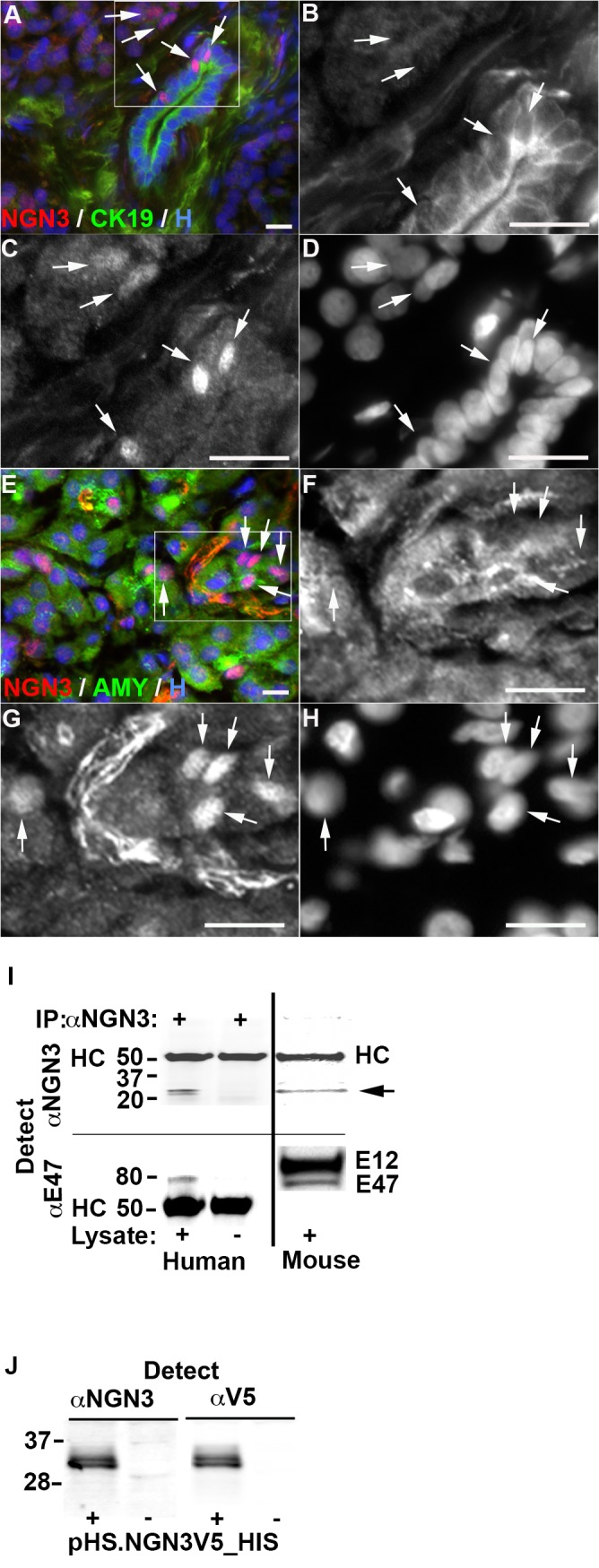
Expression of neurogenin 3 (NGN3) in the adult human exocrine pancreas. **A-H**, Immunohistochemical staining of histologically normal tissue from living subjects undergoing medically indicated pancreas biopsy using anti-NGN3 antibody F25A1B3. **A-D**, Expression of NGN3 and cytokeratin 19 (CK19) in by duct cells. B-D, Higher magnification of tissue shown in A. **B**, CK19 expression, **C**, NGN3 expression, **D**, Hoechst 33342 stained nuclei (H). **E-H**, Expression of NGN3 and amylase (AMY) by acinar cells. F-H, Higher magnification of tissue shown in E. **F**, Amylase expression, **G**, NGN3 expression, **H**, Hoechst 33342 stained nuclei. NGN3+ cells indicated by white arrowheads. Scale bars are 20 μm. **I**, Immunoprecipitation (IP) of NGN3 and E12/47 from human exocrine tissue after 4 days of culture. Presence of IP antibody F25A1B3 shown on top. F25A1B3 and anti-E12/47 detection antibodies shown on left. Presence of human or E14.5 mouse pancreatic epithelia lysate shown on bottom. Detection with anti-NGN3 (top panel) reveals bands in human and mouse lysates corresponding to the predicted molecular mass of NGN3 (~23KDa, arrow) and capture antibody heavy chain (HC). Detection with anti-E12/47 (bottom panel) identifies coimmunopreciptated proteins corresponding in size to E12 and E47. Molecular weight markers shown at left of blots in kDa. **J**, HEK293T cell lysate expressing human NGN3 tagged with V5 and 6xHIS epitopes (pHS.NGN3V5_HIS) (+) or negative control vector (-) detected with NGN3 antibody F25A1B3 and anti-V5 as indicated. Molecular weight markers shown at left of blots in KDa.

NGN3 protein expression was detected in grossly and histologically normal tissue from surgically resected pancreata taken from living subjects undergoing medically indicated pancreas biopsy. A mean ± SEM of 2.4 ± 1.1% (n = 5) of cells were NGN3+ using a primary antibody to mouse NGN3 (F25A1B3). NGN3 protein was localized in the nucleus of cytokeratin 19 (CK19)+ duct cells and amylase (AMY)+ acinar cells (representative images in Fig 1A–1H and S1A–S1P Fig). No expression of NGN3 could be detected within insulin C-peptide (CPEP)+ or chromogranin A (CHGA)+ islets. Although most of these biopsies were from normal regions of pancreata with some underlying pathology, tissue #159 (summary of biopsies shown in S1 Table) was biopsied due to splenic invagination and may reflect NGN3 expression in otherwise normal pancreatic tissue. NGN3 similarly was restricted to exocrine cells and expressed in 10.2 ± 0.5% (n = 4) of cells in biopsies taken from cadaveric pancreata 4–12 hours after organ removal (representative images in S1Q–S1T Fig). No significant difference in the percentage of NGN3+ cells was observed in these cadaveric biopsies using a primary antibody to human NGN3 (11.1 ± 0.5%, p = 0.23, n = 4).

To confirm interspecies specificity, F25A1B3 was used to detect NGN3 from mouse and human sources. Two bands immunoprecipitated from human exocrine tissue lysate migrated at the predicted mass of NGN3 and were coincident with NGN3 detected from E14.5 mouse pancreatic epithelial lysates (Fig 1I, top right panel). E2A protein E47, which forms heterodimers with NGN3 to regulate proendocrine gene transcription [44] coprecipitated with human NGN3 and was detected along with E12, an alternately spliced form of E2A, in the mouse lysate (Fig 1I, bottom right panel). The cross-reactivity of antibody F25A1B3 with human NGN3 was established further by binding to V5 epitope-tagged human NGN3 expressed in HEK293T cells (Fig 1J).

### The Percentage of Cells Expressing NGN3 Protein Increases during Exocrine Culture

**Fig 2:**
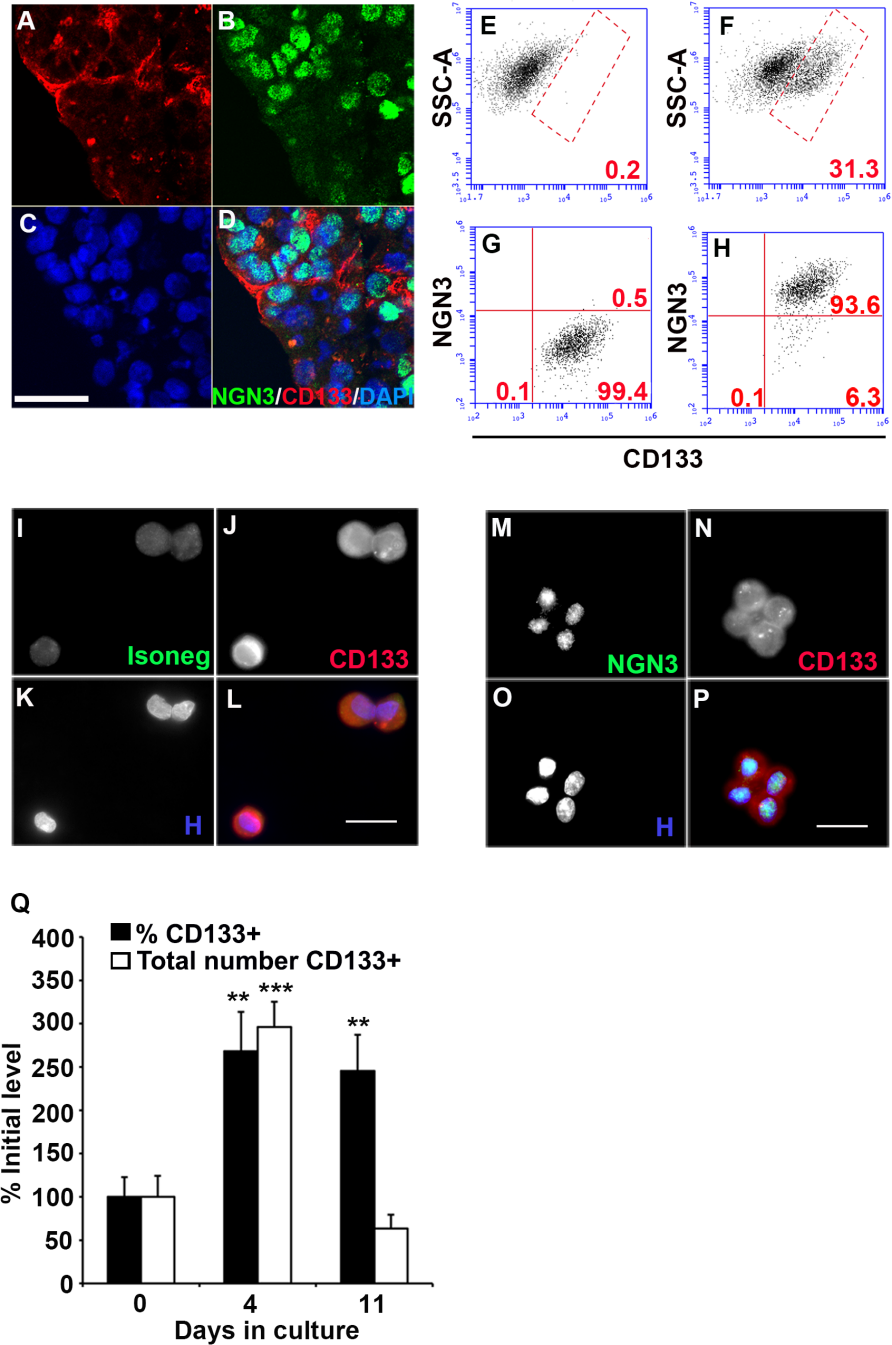
Coexpression of neurogenin 3 (NGN3) and CD133 in cultured human exocrine tissue. **A-D**, Expression of NGN3 and CD133 in exocrine tissue. **A**, CD133 expression. **B**, NGN3 expression. **C**, Nuclei costained with 4',6-diamidino-2-phenylindole (DAPI). **D**, Overlay of 3 channels. 1-μm confocal sections. Scale bar is 50 μm. **E-H**, FACS analysis of exocrine cells after 4 days in culture. Gates indicated by red lines. % cells in each gate shown in red. **E**, CD133+ gate defined by isotype negative control. **F**, CD133+ cells following anti-CD133 stain. **G**, Cells within the CD133+ gate following staining with NGN3 isotype negative control. **H**, Cells within the CD133+ gate following staining with anti-NGN3. **I-P**, Parallel fluorescence microscopy imaging of cell populations in E-H. **I**, Cells stained with NGN3 isotype negative control. **J**, Cells stained with anti-CD133. **K**, Cells stained with Hoechst 33352. **L**, Overlay of images in I-K. **M**, Cells stained with anti-NGN3. **N**, Cells stained with anti-CD133. **O**, Cells stained with Hoechst 33352. **P**, Overlay of images in M-O. Scale bars are 20 μm. **Q**, Change in the percentage and total number of CD133+ cells over time in culture. Mean ± SEM percentage of CD133+ cells (black bar) and total number of CD133+ cells (white bar) indicated along Y-axis as a percentage of initial level on day 0 of culture. Significance determined by ANOVA with Bonferroni-Holm post hoc analysis, ***, P<0.001, **, P<0.01, (n = 6 exocrine cultures).

To investigate the regulation of NGN3, living exocrine tissue was obtained from adult human pancreata that had been enzymatically digested and processed to remove islets for allogeneic islet transplantation [45, 46]. The mean (n = 5) percentage of cells expressing NGN3 protein increased significantly after culture for four days in serum-free media compared to initial levels. Although expression of NGN3 mRNA was detected in all cultures, it increased significantly only in two of five cultures and decreased significantly in one of five cultures (Table 1, representative image of NGN3 protein expression after 4 days of culture in Fig 2B). The mean (n = 3) percentage of cells expressing NGN3 protein also increased significantly in exocrine cultures maintained for four days in serum-containing media.

**Table 1:** Neurogenin 3 (NGN3) expression in cultured exocrine tissue.

		Normalized NGN3 mRNA			% NGN3+ Nuclei		
Sample	Day	Mean ± SEM	% Change	p	Mean ± SEM	% Change	p
A	2	7.1 ± 0.2			12.2 ± 2.8		
	6	11.7 ± 0.3	164.8	<0.01	28.4 ± 9.4	232.8	<0.05
B	3	2.1 ± 0.1			1.6 ± 1.1		
	7	2.1 ± 0.1	100.0	>0.05	6.4 ± 1.3	400.0	<0.05
C	3	23.8 ± 0.4			7.4 ± 4.1		
	7	12.4 ± 0.3	52.1	<0.001	46.2 ± 4.2	624.3	<0.001
D	2	1.1 ± 0.1			13.2 ± 3.5		
	6	4.7 ± 0.2	427.3	<0.001	52.6 ± 10.2	398.5	<0.001
E	3	1.3 ± 0.1			0.5±0.2		
	7	1.6 ± 0.1	123.1	>0.05	13.3±4.1	2660.0	<0.05
Mean			173.4			863.1	
SEM			66.0			453.5	

Neurogenin 3 (NGN3) expression in cultured exocrine tissue from five cadaveric pancreata coded as sample A-E. Day, Day post mortem tissue was processed for analysis. Mean ± SEM NGN3 mRNA level calculated from 6 readings. NGN3 mRNA level normalized to level of cyclophillin A. Mean ± SEM %NGN3+ nuclei calculated from 10 fields. % Change, Change in level of expression over 4 days of culture (e.g. day 2 vs. day 6). Mean ± SEM of all five samples shown at bottom. Significance (p) determined by ANOVA with Bonferroni-Holm post hoc analysis.

The basis for an increased percentage of cells expressing NGN3 protein was investigated. Cell proliferation was excluded due to a lack of incorporation of nucleoside analog 5-ethynyl-2'-deoxyuridine (EdU) over four days of culture (n = 3). Selective NGN3-negative cell death was ruled out based on the low level of cell death observed in exocrine tissue at receipt (1.6 ± 0.5% TUNEL labeled cells (n = 5)) and after culture (6.4 ± 0.8% TUNEL labeled cells (n = 5)). A third possibility, *de novo* expression of NGN3 expression by exocrine cells, is suggested by the global shift in exocrine cell gene expression that occurs following pancreas injury and culture. This acinar-to-ductal metaplasia is characterized by the loss of mature exocrine cell functional proteins such as AMY and the gain of a duct-like phenotype characterized by expression of CK19 [47–49]. In exocrine cultures, AMY expression decreased significantly from a mean ± SEM of 37.1 ± 5.2% of cells on receipt to 2.7 ± 1.3% of cells after four days (p<0.001, n = 3). Expression of CK19 increased significantly from 35.4 ± 3.9% to 64.0 ± 7.0% over this period (p<0.01, n = 3). After culture, virtually all NGN3+ nuclei were localized in CK19+ cells.

### NGN3 Is Coexpressed with CD133

The cell surface glycoprotein CD133, encoded by the prominin 1 gene (PROM1), is expressed on the apical surface of carbohydrate antigen 19–9+ pancreatic ductal epithelial cells, as well as by stem and progenitor cells in a variety of tissues [50–54]. CD133 has been shown to be coexpressed with NGN3 protein in the human fetal pancreas [55] and NGN3 mRNA is expressed by CD133+ cells isolated from the adult human pancreas [56].

After culture, CD133 immunoreactivity is not restricted to the ductal lumen and is associated with NGN3+ nuclei (Fig 2A–2D). FACS analysis of cultured exocrine tissue costained for CD133 and NGN3 indicated a mean ± SEM (n = 2) of 94.3 ± 3.5% of CD133+ cells positive for NGN3 (representative FACS shown in Fig 2E–2H). Based on this high degree of coexpression, immunomagnetic cell sorting for CD133 was used to prepare highly enriched NGN3 positive (CD133+, >95% CD133+) and NGN3 negative (CD133-depleted, <1% CD133+) cell populations from exocrine cultures. Imaging of >1000 CD133+ cells sorted and stained in parallel to the FACS analysis demonstrated the expected membrane/cytoplasmic and nuclear patterns for CD133 and NGN3, respectively (representative images in Fig 2I–2P). Every cell examined indicated the coexpression of both proteins.

The mean (n = 6) percentage and total number of cells expressing CD133 increased significantly after culture relative to initial levels (Fig 2Q), consistent with observed increases in NGN3 protein expression. The increase in total number of CD133+ cells further suggests an increase in NGN3 protein expression by cells that previously were NGN3-negative rather than selective loss of NGN3-negative cells, as the latter could not result in an increase in total number.

### The CD133+ Cell Population Does Not Contain Beta Cells

Islet-depleted exocrine tissue, a byproduct of allogeneic islet transplantation, was used as a source of human pancreas tissue in order to minimize the possibility that mature endocrine cells were present in isolated CD133+ cell populations. Few islets or beta cells were detected in exocrine cultures stained with dithizone, a zinc-binding stain used to visualize insulin (mean ± SEM 0.01 ± 0.01% cells (n = 5)) on receipt and no dithizone staining could be detected after four days in culture (n = 5). Islets that dedifferentiate in culture can lose insulin expression but retain expression of CHGA [57]. Isolated human islets (~90% islets) were cultured for four days under the same condition used for exocrine tissue to determine if dedifferentiated islet cells expressed CD133. Immunohistochemical staining for CD133 with CPEP or CHGA demonstrate a clear segregation between islets and CD133+ exocrine tissue surrounding islets (representative image shown in S3 Fig). The absence of beta cells in CD133+ cell populations was routinely confirmed using quantitative PCR assays specific to insulin and CHGA.

The presence of CD133 was used to isolate NGN3+ cells for expression profiling and *in vitro* endocrine differentiation on the basis of coexpression of NGN3 and CD133, significant enrichment of NGN3 mRNA in CD133+ cells compared to the CD133D population (196.2 ± 40.9-fold, p<0.01, n = 4) and the absence of preexisting beta cells following selection. After four days in culture, the mean ± SEM CD133+ cell yield was 1.4X10^7^ ± 4.0X10^6^ CD133+ cells/ml of pelleted exocrine tissue (n = 10). The CD133+ / NGN3+ cell population is designated hereafter as CD133+ when describing experiments that directly involved cell isolation and as NGN3+ when describing general properties of the population.

### The CD133+ Cell Transcriptome Differs Significantly from Surrounding Exocrine Cells

Transcriptome sequencing of CD133+ and CD133D populations was carried out to investigate the identity and cell fate potential of NGN3+ cells. Comparisons of these populations reveal significant differential expression in gene, isoform, coding sequence (CDS) and transcriptional start site. The top 20 differentially expressed transcripts in each transcript category are listed in S2 Table. Cystic fibrosis transmembrane conductance regulator (CFTR), which is expressed by many types of epithelial cells including pancreatic ducts, is among the most highly overexpressed genes in CD133+ cells compared to CD133D, as is matrix metalloproteinase-7 (MMP7), a matrix endopeptidase known to breakdown extracellular matrix components and activate membrane bound receptors such as Notch [58]. Although PROM1 was highly overexpressed in the CD133+ population, NGN3 and many other transcription factors were insufficiently represented in the transcriptome analyses to calculate relative expression levels. In addition to transcript level differences, significant differential splicing between isoforms processed from a single primary transcript, differential CDS output from multi-protein genes and differential promoter usage between the CD133+ and CD133D populations also were observed (S3 Table). Differential promoter usage of PROM1, known to have multiple promoters and alternatively spliced isoforms [59], was detected.

Functional annotation was used to identify classes of genes significantly overexpressed by the CD133+ population compared to CD133D. Significant differential expression was detected in several different categories including transcriptional regulators, receptor proteins, cytokines, genes expressed during endocrine development, genes associated with bone morphogenetic protein, Wnt and Notch signaling, as well as in genes correlated with type 2 diabetes [60].

Transcriptional analysis also identified a number of highly expressed genes, defined here as >1000 fragments per kilobase of transcript per million mapped fragments (FPKM). Two genes commonly used as endogenous expression controls, cyclophillin A and glyceraldehyde 3-phosphate dehydrogenase, had expression levels of ~100 and ~2500 FPKM, respectively. Regenerating islet-derived 1A (REG1A), the most highly expressed gene in CD133+ cells (41,985 FPKM) has been associated with islet regeneration [61] and like REG1B which is also highly expressed by CD133+ cells, is transcribed at high levels by cells of the endocrine and exocrine pancreas. The exocrine functional proteins pancreatic secretory trypsin inhibitor (SPINK), trypsin-1 and -2 (PRSS1, 2) as well as alpha 1-antitrypsin 3 (SERPINA3) are highly expressed by CD133+ cells and are expressed at even higher levels by the CD133D population.

### NGN3 Is Negatively Regulated by Notch Signaling

**Fig 3:**
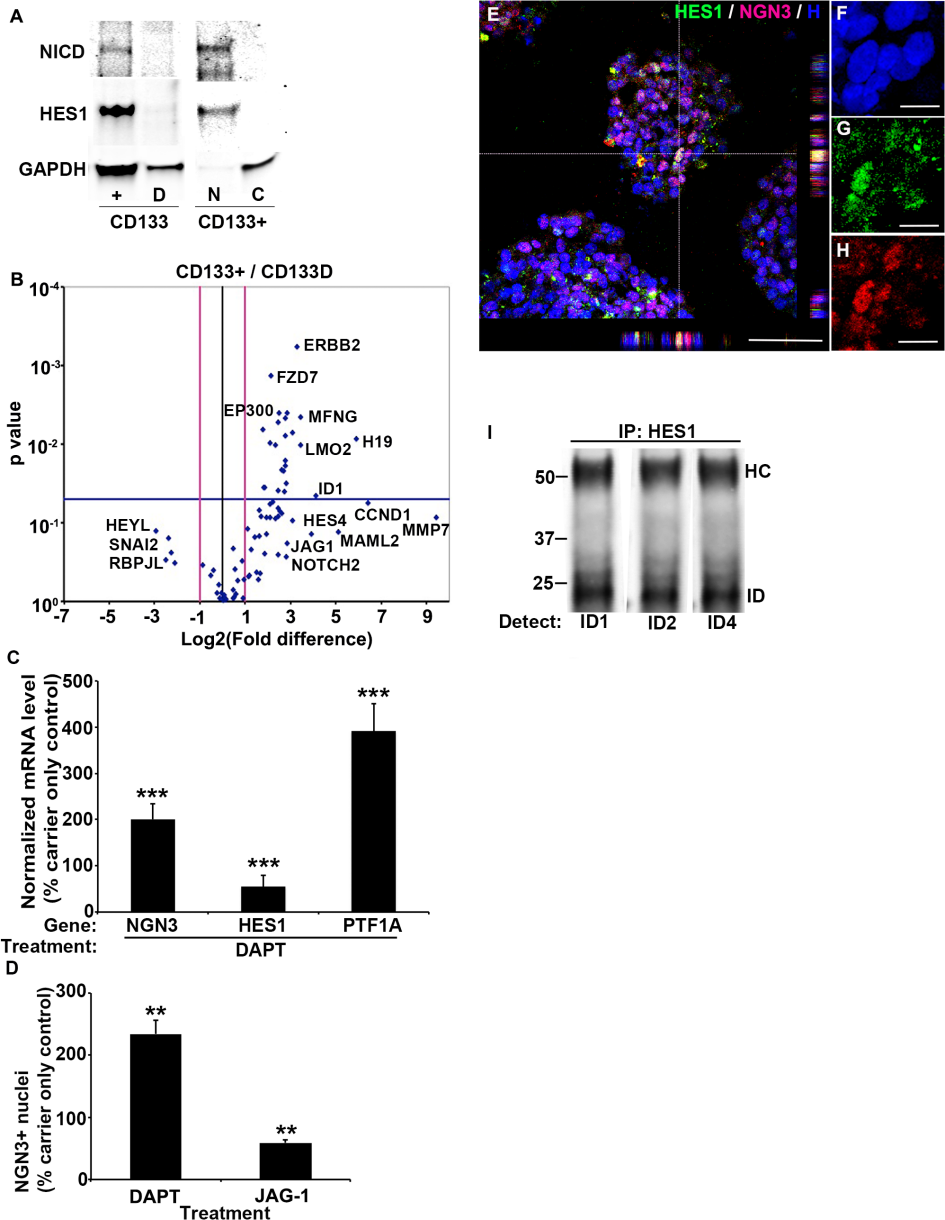
Expression of Notch pathways genes. **A**, Western blot analyses of Notch intercellular domain (NICD), hairy enhancer of split 1 (HES1) and endogenous control gene glyceraldehyde-3-phosphate dehydrogenase (GAPDH) in cells isolated from human exocrine tissue. Whole cell lysates from CD133+ (+) and CD133-depleted (D) cells. Nuclear (N) and cytoplasmic (C) extracts from CD133+ cells. **B**, Volcano plot of Notch pathway gene mean ± SEM mRNA level (n = 3 exocrine cultures) differences in expression level from CD133+ cells compared to CD133D shown on X-axis as Log2 of fold difference. Significance determined by Student’s t-test shown on Y-axis as p value. Magenta vertical lines mark a 2-fold difference in expression. Blue horizontal line marks the significance cutoff (p<0.05). Selected gene names shown. Genes are: receptor tyrosine-protein kinase erbB-2 (ERBB2), frizzled class receptor 7 (FZD7), E1A binding protein p300 (EP300), MFNG O-fucosylpeptide 3-beta-N-acetylglucosaminyltransferase (MFNG), H19, imprinted maternally expressed transcript (H19), LIM domain only 2 (rhombotin-like 1) (LMO2), inhibitor of DNA binding 1 (ID1), hairy enhancer of split 4 (HES4), cyclin D1 (CCND1), matrix metallopeptidase 7 (MMP7), mastermind-like 2 (MAML2), jagged 1 (JAG1), Notch 2 (NOTCH2), hes-related family bHLH transcription factor with YRPW motif-like (HEYL), snail family zinc finger 2 (SNAI2), recombination signal binding protein for immunoglobulin kappa J region-like (RBPJL). **C**, Normalized mRNA expression level of neurogenin 3 (NGN3), HES1 and pancreas transcription factor 1 subunit alpha (PTF1A) in exocrine tissue after 4 days of culture in the presence of 20 μM Notch inhibitor DAPT. Results reported as mean ± SEM percent of levels in DMSO carrier control. mRNA levels normalized to the level of cyclophillin A. Significance determined by Student’s t-test, ***, p<0.001 (n = 3 exocrine cultures). **D**, Expression of NGN3 protein following treatment with 20 ∞M DAPT and 47 ∞M Notch agonist JAG-1 peptide (JAG-1). Mean ± SEM percent of DMSO carrier only control or 47 ∞M scrambled JAG-1 peptide, respectively indicated on Y-Axis. Significance determined by Student’s t-test, ***, p<0.001 (n = 3 exocrine cultures). **E-H**, Orthogonal analysis of colocalized HES1 and NGN3 in nuclei of exocrine tissue after 4 days of culture. Nuclei counterstained with Hoechst 33342 (H). **E**, Overlay of 3 channels. 0.5 ∞m confocal section. Scale bar is 50 ∞m. **F-H**, Higher magnification of crosshair region in all three channels shown at right. Scale bars are 20 ∞m. **I**, Coprecipitation of ID proteins with HES1. Whole cell lysate from exocrine tissue after 4 days of culture immunoprecipitated with antibody to HES1. ID1, 2 and 4 detected following SDS PAGE and western blotting. Predicted molecular weights of ID proteins (ID) and immunoglobulin heavy chain used for precipitation (HC) shown at right. Molecular weight marker positions shown at left in kDa.

During pancreas development, signaling by the Notch receptor plays an essential role in maintaining the NGN3+ endocrine progenitor population through activation of the transcriptional repressor HES1 [1, 5, 62–69]. Notch regulation of NGN3 was investigated to determine if it plays a similar role in the adult pancreas. Notch signaling results in proteolytic cleavage and nuclear translocation of the Notch intercellular domain (NICD). Both NICD and HES1 were detected in whole cell and nuclear extracts of CD133+ but not CD133D cells (Fig 3A). Furthermore, mRNA from CD133+ and CD133D cell populations were screened with a panel of genes involved in Notch signaling, Notch downstream targets and pathways that crosstalk with Notch. Of 59 genes with >2-fold changes in mean (n = 3) expression, 54 are upregulated in the CD133+ cell population compared to CD133D (Fig 3B). Expression of all four Notch genes as well as ligands jagged (JAG1, 2) and delta-like (DLL1, 4) also were detected in CD133+ and CD133D transcriptomes.

To determine if Notch signaling is actively regulating expression of downstream genes in exocrine tissue, the mean (n = 3) mRNA expression levels of HES1, NGN3 and pancreas-specific transcription factor 1A (PTF1A) were measured following four days of Notch inhibition with DAPT, a gamma-secretase inhibitor, which prevents Notch cleavage [70]. HES1 mRNA decreased significantly in the presence of DAPT, consistent with transcriptional activation by Notch. NGN3 and PTF1A mRNAs, which are regulated negatively by Notch signaling [65, 71], increased significantly (Fig 3C) as did the mean (n = 3) percentage of cells expressing NGN3 protein (Fig 3D). Conversely, the mean (n = 3) percentage of cells expressing NGN3 protein decreased significantly in the presence of Notch agonist, JAG-1 peptide compared to cells treated with an equal concentration of a negative control peptide comprised of scrambled JAG-1 amino acids (Fig 3D).

The presence of HES1 in CD133+ protein extracts was unexpected given the repressive role of HES1 on NGN3 transcription and their segregation during murine fetal development [72]. This observation was supported by NGN3 and HES1 colocalization in the nuclei of 10.2 ± 1.8% (n = 3) of all cells and 79.1 ± 6.4% (n = 3) of HES1+ nuclei of cultured exocrine tissue (representative image in Fig 3E–3H). Colocalization of HES1 and NGN3 in adult exocrine tissue suggests neutralization of HES1 repression, possibly through the inhibitor of DNA binding proteins (ID1-4), which can form heterodimers with HES1 to block transcriptional regulation [73, 74] and are significantly upregulated in CD133+ cells. Coimmunopreciptation of ID1, ID2 and ID4 with HES1 is consistent with their role in neutralization of HES1 repression (Fig 3I).

### CD133+ Cells Express Transcription Factors Characteristic of Early Endocrine Progenitors

**Fig 4:**
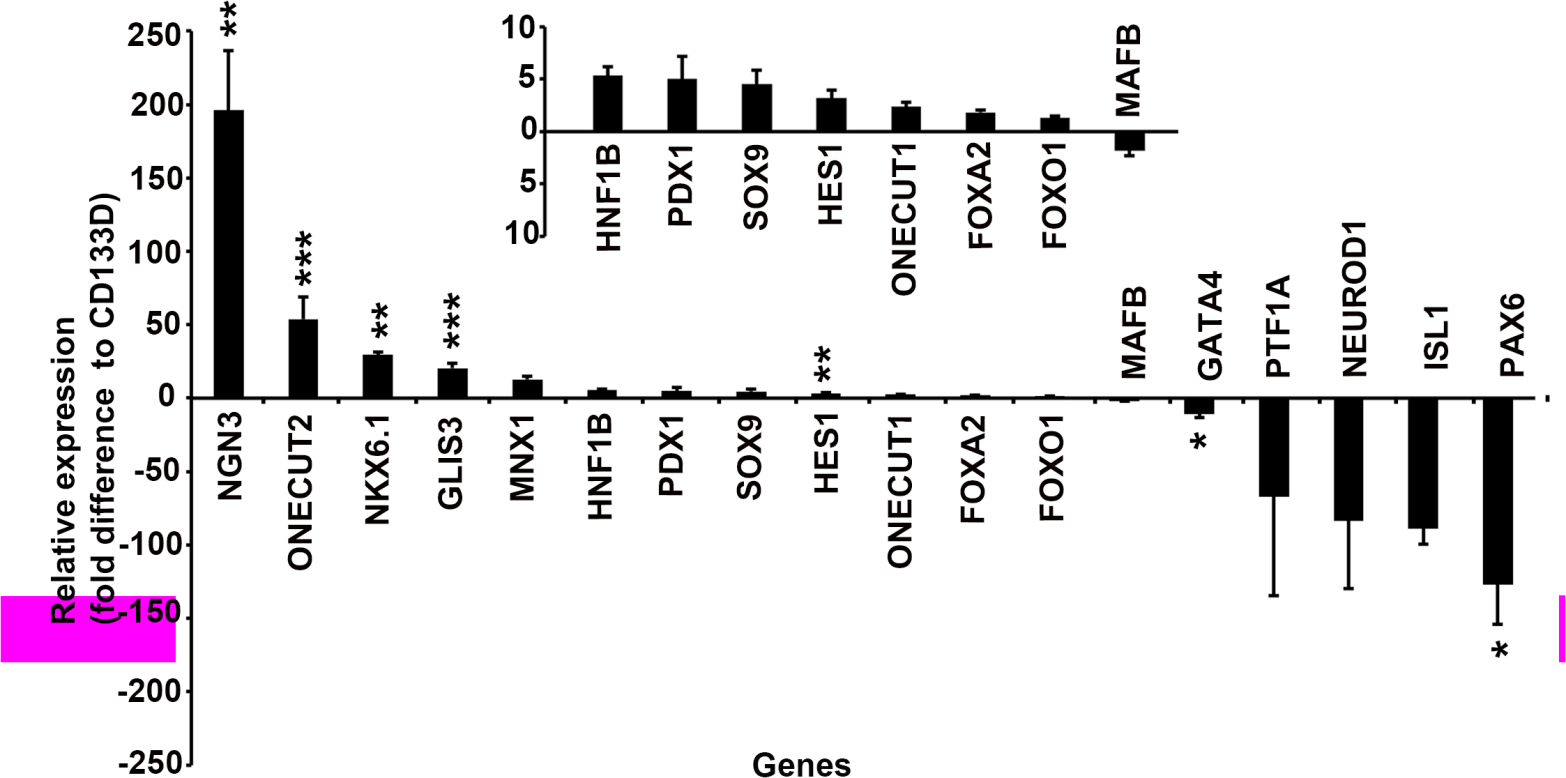
Relative mRNA expression of pancreas development transcription factors by CD133+ cells isolated after 4 days of exocrine tissue culture. Mean ± SEM relative expression level of genes in CD133+ cells compared to the CD133-depleted (CD133D) population shown on Y-axis as fold difference. Significance determined by Student’s t-test from 2^-Δct^ values. ***, p<0.001, **, p<0.01, *, p<0.05 (n = 4 exocrine cultures). Genes ranked in order of overexpression in the CD133+ population. Inset shows gene expression using an enlarged scale. Genes are: Neurogenin 3 (NGN3), One cut homeobox 2 (ONECUT2), NK6 homeobox 1 (NKX6.1), GLIS family zinc finger 3 (GLIS3), motor neuron and pancreas homeobox 1 (MNX1), HNF1 homeobox B (HNF1B), pancreatic and duodenal homeobox 1 (PDX1), SRY (sex determining region Y)-box 9 (SOX9), Hairy enhancer of split 1 (HES1), One cut homeobox 1 (ONECUT1), Forkhead box A2 (FOXA2), Forkhead box O1 (FOXO1), v-maf avian musculoaponeurotic fibrosarcoma oncogene family, protein B (MAFB), GATA binding protein 4 (GATA4), Pancreas specific transcription factor 1A (PTF1A), Neuronal differentiation 1 (NEUROD1), ISL LIM homeobox 1 (ISL1), paired box 6 (PAX6). No expression of NK2 homeobox 2 or paired box 4 was detected.

Endocrine pancreas development proceeds through expression of a set of transcription factors that enforce lineage restriction and stabilize mature cell phenotypes. The mean (n = 4) relative mRNA expression of 20 endocrine developmental transcription factors was used to characterize CD133+ cells by comparison to stages of murine endocrine development. As expected, NGN3 was significantly enriched in CD133+ cells compared to CD133D, along with onecut homeobox 2 (ONECUT2), NK6 homeobox 1(NKX6.1), GLIS family zinc finger 3 (GLIS3) and HES1. PTF1A and neuronal differentiation 1 (NEUROD1), transcription factors required for mature exocrine and endocrine function, respectively, were both under expressed by CD133+ cells, as were GATA binding protein 4 (GATA4), paired box 6 (PAX6) and ISL LIM homeobox 1 (ISL1) (Fig 4). Expression of paired box 4 (PAX4) and NK2 homeobox 2 (NKX2.2) was not detected in either population.

### Differentiating NGN3+ Cells Express Markers of Endocrine Development and Islet Hormones

**Fig 5:**
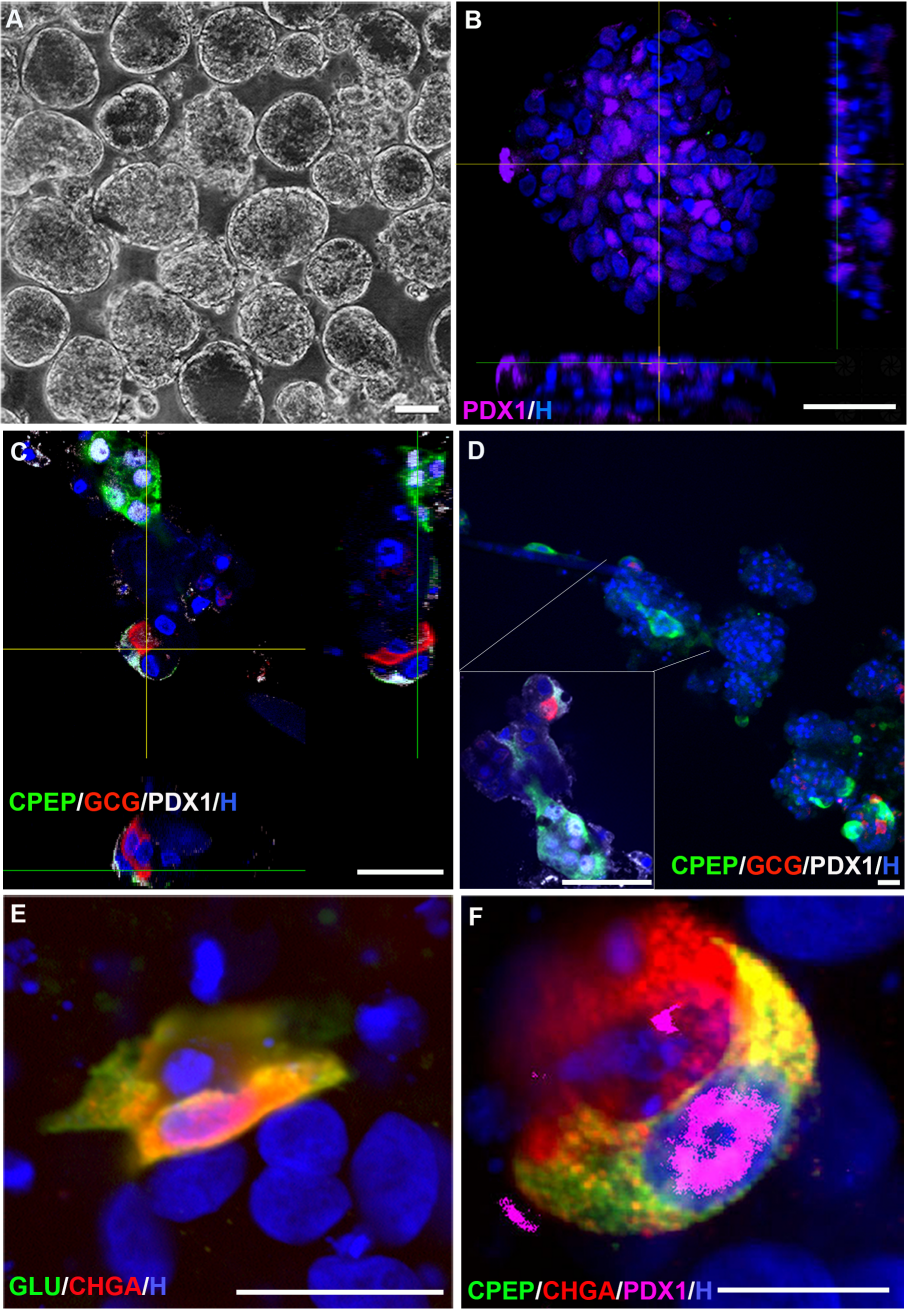
Expression of hormones, chromogranin A and PDX1 by CD133+ cells following *in vitro* differentiation. **A**, Phase microscopic image of pancospheres on day 6 of formation. Scale bar is 100 μm. **B**, Pancreatic and duodenal homeobox 1 (PDX1) expression in a day 6 pancosphere. Scale bar is 50 μm. **C,D**, Orthogonal analyses of PDX1 / glucagon (GCG) and PDX1 / insulin C-peptide (CPEP) coexpression in day 21 pancospheres. 1 μm optical sections, scale bar is 50 μm. Inset box in D is magnified and rotated confocal reconstruction of cells indicated by lines. **E**, Coexpression of GCG and chromogranin A (CHGA) by cells within a day 21 pancosphere. Scale bar is 20 μm. **F**, Coexpression of CPEP, CHGA and PDX1 by cells within a day 21 pancosphere. Scale bar is 10 μm. B-F, Nuclei stained with Hoechst 33342 (H).

CD133+ cells were differentiated *in vitro* to test their capacity to attain an endocrine cell phenotype. Suspension culture of CD133+ cells in media conditioned with human SDEC cells [75] results in spherical cell aggregates termed pancospheres (PS), which resemble structures that form following suspension culture of aldehyde dehydrogenase positive mouse pancreatic cells [76](representative image Fig 5A). Attempts to form PS in non-conditioned base media, media conditioned with other cell lines or in defined media used to derive neurospheres from human fetal brain CD133+ cells [77] failed to produce PS in sufficient quantity for analysis. Antibody array analysis of SDEC cell conditioned media identified expression of 70 cytokines (S4 Table), some of which also were identified in microarray comparisons of SDEC cells to other human cell types [78]. After 6 days of formation (PS day 6), PS had a mean ± SEM diameter of 162 ± 4.7 microns (n = 300 PS) and 30.8 ± 7.6% of cells were EdU+ (n = 16 PS). Additionally, 32.3 ± 3.6% (n = 12 PS) of cells were NGN3+ and virtually all cells expressed E-cadherin+. CPEP+ or glucagon (GCG)+ cells were not detected at this stage. 12.1 ± 2.7% of single CD133+ cells formed clonal PS (n = 3), whereas CD133D cells failed to form PS even when plated at 100 cells per well (n = 2). PS at this stage can be passaged 3–4 times with a combination of trypsin and mechanical disaggregation.

**Fig 6:**
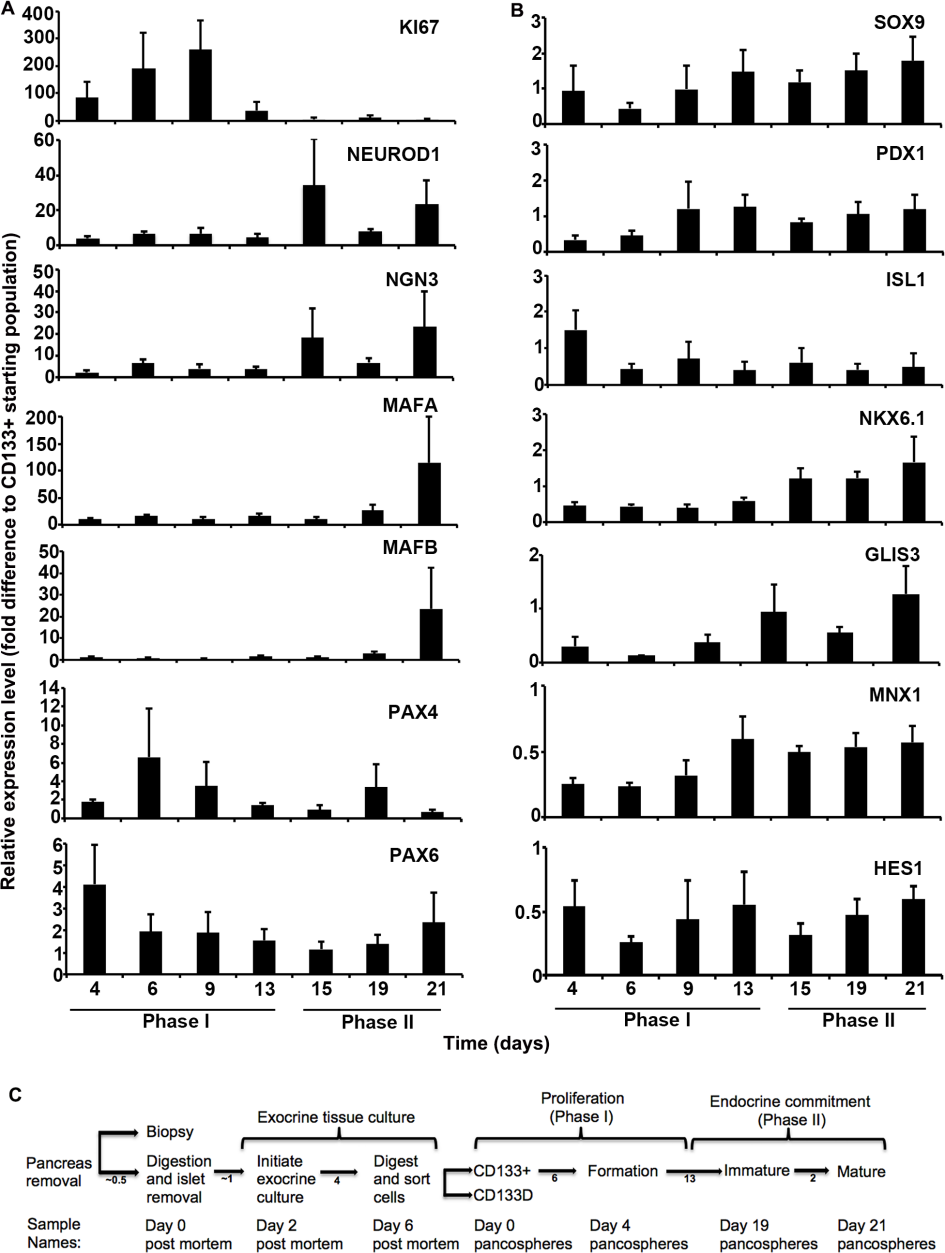
Relative mRNA expression level of endocrine development transcription factors by differentiating pancospheres (PS) over time. Mean ± SEM (n = 3 exocrine cultures) relative expression level reported as fold difference from CD133+ starting population shown on Y-axis. Days after initiating PS formation shown along X-axis. Proliferative (Phase I) and maturation (Phase II) phases shown at bottom. **A**, Upregulated genes are: marker of proliferation Ki-67 (KI67), Neuronal differentiation 1 (NEUROD1), Neurogenin 3 (NGN3), v-maf avian musculoaponeurotic fibrosarcoma oncogene homolog A (MAFA), v-maf avian musculoaponeurotic fibrosarcoma oncogene family, protein B (MAFB), paired box 4 (PAX4), paired box 6 (PAX6). **B**, Down regulated genes are: SRY (sex determining region Y)-box 9 (SOX9), pancreatic and duodenal homeobox 1 (PDX1), ISL LIM homeobox 1 (ISL1), NK6 homeobox 1 (NKX6.1), GLIS family zinc finger 3 (GLIS3), motor neuron and pancreas homeobox 1 (MNX1), Hairy enhancer of split 1 (HES1). **C**, Isolation and endocrine differentiation of NGN3+ cells. After death, pancreas is removed and transported to an islet isolation facility where a biopsy is taken for histology. The pancreas is then digested and separated into islets and exocrine tissue. Exocrine culture is initiated on day 2 post mortem. On day 6 post mortem, tissue is digested to single cells, which are labeled and sorted for expression of CD133. CD133+ cells are plated on pancosphere day 0. Samples are collected on pancosphere days 4, 6, 9, 13, 15, 19 and 21. Cell proliferation marker KI67 expression peaks at pancosphere day 9. On pancosphere day 19 IGF II is withdrawn and cells begin final maturation. Days between each step are indicated below arrows. Exocrine tissue culture and phases of pancosphere differentiation are shown above the timeline.

Over 21 days, PS undergo two distinct phases of differentiation (Fig 6). During Phase I (PS day 4–13), PS cells proliferate as indicated by EdU incorporation, increased mean ± SEM diameter of 824 ± 23.9 microns on PS day 15 (n = 20 PS) and a high mean mRNA expression of KI67 compared to the CD133+ starting population (n = 3 for all genes profiled). Phase II (PS day 15–21) is marked by decreased expression of KI67 and increased expression of NEUROD1, a factor that regulates terminal differentiation and mature function of islet cells [79, 80]. NGN3, which positively regulates NEUROD1 in developing endocrine cells [81, 82], is expressed in a similar pattern to NEUROD1. The expression of MAFA and v-maf avian musculoaponeurotic fibrosarcoma oncogene B (MAFB), which are required for beta cell function [83, 84], peak after the final step of differentiation. Although the overall expression level of PAX4 was relatively low throughout differentiation, it had a bimodal expression pattern with peaks of expression on PS day 6 and 19.

Expression of beta and alpha cell proteins PDX1, CPEP, GCG and CHGA was investigated to characterize the final stages of endocrine differentiation. The percentage of PS cell nuclei positive for PDX1 significantly decreased 16.5-fold (p<0.001, n = 3) between PS formation on PS day 6 (Fig 5B) and final maturation on PS day 21 (Fig 6C–6F). Mycophenolic acid, an inhibitor of GTP synthesis shown to promote endocrine development in zebrafish [85], significantly increased the percentage of CPEP+ and GCG+ cells 6.9 and 8.4 fold (p<0.05, n = 3), respectively (representative images in Fig 5C and 5D). Although CPEP+ and GCG+ cells often were present in the same PS, coexpression of CPEP and GCG was not detected. Virtually all CPEP+ and GCG+ cells coexpressed CHGA and approximately 77% CPEP+ / CHGA+ cells expressed PDX1 localized in the nucleus (n = 15 PS) (representative image shown in Fig 6F).

## Discussion

The presence of NGN3 protein in the adult human pancreas differs from expression in adult rodents, where it only is detected transiently and following specific types of injury or experimental manipulation. NGN3 protein has been identified here in surgical and cadaveric biopsies of histologically normal adult human pancreata. The difference between NGN3 expression in freshly isolated surgical biopsies and cadaveric biopsies may be due to changes in expression known to occur rapidly after pancreas removal [86], individual variation of NGN3 expression or other variables associated with tissue processing.

Significant differences in gene expression, gene splicing, promoter usage, protein CDS output and Notch activity suggest adult NGN3+ cells are distinct from surrounding exocrine tissue. During development, NGN3 is expressed by endocrine progenitor cells maintained in a proliferative state prior to final cell fate commitment. In the adult human pancreas, ubiquitous distribution within acini and ducts, as well as expression of genes characteristic of exocrine function, suggest NGN3+ cells were mature exocrine cells that underwent dedifferentiation rather than a specialized population of endocrine progenitor cells, centroacinar cells or dedifferentiated endocrine cells. Coexpression of CD133 by the NGN3+ population further supports this possibility as it has been associated with epithelial dedifferentiation [54], as does the increase in the percentage and total number of NGN3+ cells during culture of exocrine tissue in the absence of sufficient proliferation or selective cell death.

We sought to determine if Notch regulated NGN3 in the adult pancreas due to its central role regulating NGN3 transcription and protein stability [72] during pancreas development. The presence of NICD, upregulation of Notch target gene expression and pharmacological modulation of NGN3 expression in culture all suggest Notch signaling actively regulates NGN3 transcription and protein levels. However, Notch repression of NGN3 through HES1 is attenuated, possibly through the observed formation of HES1-ID protein dimers. The disparity between changes in NGN3 mRNA and protein levels following culture suggests both transcriptional and post-transcriptional pathways regulate NGN3 in adult exocrine tissue.

When compared to stages of murine endocrine development, the expression phenotype of isolated human NGN3+ cells most closely matches murine E12.5 early endocrine progenitor cells [87, 88]. Both cell population express hepatocyte nuclear factor 1 (HNF1), ONECUT1 and 2, PDX1, NKX6.1, SRY box 9 (SOX9), NGN3 and GLIS3, but low levels or no PAX6, ISL1, MAFB or NEUROD1. Although murine E12.5 cells express PAX4 and NKX2.2, failure to detect expression of these genes in NGN3+ cells prior to differentiation is consistent with their absence in human 47–52 day post conception NGN3+ cells [6–8], a population which otherwise corresponds to murine E12.5 endocrine progenitors. In addition to an endocrine progenitor phenotype, NGN3+ cells undergo a pattern of gene expression that resembles fetal endocrine development. Although the efficiency of *in vitro* endocrine differentiation in PS is low, it results in cells expressing MAFA and MAFB mRNA and a protein expression phenotype (CPEP+ / CHGA+ / PDX1^Nuclear^) that resembles beta cells. These mRNA and protein expression results strongly suggest NGN3+ cells from the adult human pancreas are capable of acquiring an endocrine cell fate. The role of NGN3 in this process may be to integrate pro-endocrine signals then activate target genes such as NEUROD1 to initiate cell cycle exit and endocrine fate commitment.

Although murine *in vivo* reprogramming [39, 41, 43], exogenous NGN3 expression in human exocrine cultures [29, 42, 89] and this work demonstrate exocrine cells have the capacity to acquire an endocrine cell fate after expression of NGN3, it does not necessarily imply this process occurs under normal circumstances *in vivo*. Studies demonstrating beta cell replacement in adult mice requires pre-existing beta cells [14–17] and expression of NGN3 following injury is insufficient to drive differentiation to an endocrine cell fate [36] suggest beta cell replication may be the dominant or exclusive method to maintain beta cell mass. Nevertheless, reprogramming demonstrates even subtle and transient changes to exocrine cell gene expression or cytokine milieu can establish a permissive environment for conversion to an endocrine cell fate.

Given their prevalence, it is likely that NGN3+ cells are present in autologous and some allogeneic islet transplants and may play a role in the positive correlation between the number of “ductal epithelial” cells transplanted and long-term metabolic success observed in human islet transplant recipients [90]. NGN3+ cells isolated from exocrine tissue may represent an allogeneic cell source for treatment of type diabetes and if not depleted, a potential autologous source. Pharmacological treatments targeting the negative regulation of NGN3 and its promotion of endocrine fate commitment may represent an *in vivo* therapeutic approach to islet neogenesis.

## Methods

### Pancreas Biopsies

Pancreas biopsies were obtained from living subjects undergoing medically indicated diagnostic procedures. Regions analyzed were determined to be grossly and histologically normal. Cadaveric pancreas biopsies were obtained 4–12 hrs. after organ removal (delay durations are reported in S1 Table).

### Primary Exocrine Tissue Culture

Islet-depleted exocrine tissue and purified islet preparations isolated from human pancreata of non-diabetic adult donors were received 2–3 days post mortem. In all experiments, replica number refers to the number of cultures from individual organ donors unless otherwise stated. Unless noted, exocrine tissue was resuspended in 100 ml media per 1ml of pelleted tissue in serum-free Miami Media 1A (Mediatech 98-021-CV, Manassas, VA, USA) supplemented with freshly prepared 0.01g/L reduced glutathione and maintained in suspension culture at 37°C for 4 days in low-adhesion plastic dishes. Media was replaced after 2 days. When noted, exocrine tissue was maintained in CMRL1066 media (Life Technologies, Carlsbad, CA, USA) supplemented with 10% fetal bovine serum and 2 mM L-glutamine.

Exocrine tissue at receipt and after culture was stained in freshly prepared dithizone (1mg/ml) for 20 min. room temperature then ~2000 tissue clusters were placed in each of 4 wells of a gridded bottom 6-well dish. To measure cell proliferation, 1 ml of tissue was cultured for up to 4 days in Miami Media 1A containing ≤10μM 5-ethynyl-2'-deoxyuridine (EdU, Life Technologies, Carlsbad, CA, USA). Tissue was fixed in 4% PFA, permeabilized in 0.5% Triton-X100 and EdU was detected with Alexa Fluor 488 detection reagents (Life Technologies, Carlsbad, CA, USA). Nuclei were counterstained with Hoechst 33342 then imaged using fluorescence microscopy. The level of cell death was assessed by immunohistochemistry using ApopTag assays (Millipore, Billerica, MA, USA). To investigate Notch signaling, tissue was treated with 20 μM N-[(3,5-Difluorophenyl)acetyl]-L-alanyl-2-phenyl]glycine-1,1-dimethylethyl ester (DAPT, TOCRIS Bioscience, Bristol, UK) resuspended in dimethylsulfoxide (DMSO) or with 47μM peptide corresponding to Jagged-1 amino acids 188–204 (JAG-1 peptide) or peptide with scrambled JAG-1 amino acids (Anaspec, Freemont, CA, USA) resuspended in water.

### Tissue Immunohistochemistry

Biopsy tissues were frozen and transported at ≤-80°C (biopsies) then embedded in OCT mounting media (Tissue-Tek, Sakura, Torrance, CA, USA). Cultured exocrine tissue was pelleted in OCT then snap frozen in a dry ice / 2-methylbutane bath. All tissues were stored at -80°C and sectioned as soon as possible after freezing. Fresh eight micron frozen sections were fixed for 5 min. in 4% paraformaldehyde (PFA) in phosphate buffered saline (PBS), quenched for 5 min. in 50 mM glycine in PBS then blocked in 5% donkey serum, 1% BSA, 0.1% Triton-X100 in PBS for 30 min. at room temperature. Detection of NGN3 was carried out with a mouse monoclonal antibody raised against an N-terminal epitope of mouse NGN3 (Developmental Studies Hybridoma Bank F25A1B3, hybridoma supernatant, 1:10 dilution, East Iowa City, IA, USA) or rabbit polyclonal antibody raised against an N-terminal peptide of human NGN3 (Sigma Prestige HPA039785, 1:500 dilution, St. Lois, MO, USA). F25A1B3 was used for all NGN3 staining unless specified. Additional antibodies were; mouse anti-human CA19.9 (ab3982, IgM, Abcam, Cambridge, UK), mouse anti-human CD133/2 (IgG2b, Miltenyi Biotec, Bergisch Gladbach, Germany), mouse anti-cytokeratin 19 (Chemicon, Temecula, CA, USA), rabbit anti-human insulin C-peptide (Linco Research, St. Charles, MO, USA), rat anti-human insulin C-peptide (Developmental Studies Hybridoma Bank), mouse anti-glucagon (R&D Systems, Minneapolis, MN, USA), goat anti-PDX1 (Abcam ab47383, Cambridge, UK), mouse anti-chromogranin A (Dako, Glostrup, Denmark), goat anti-amylase (Santa Cruz Biotechnology, Santa Cruz, CA, USA), rabbit anti-HES1 (Cell Signaling, Beverly, MA, USA), mouse anti-E Cadherin (Invitrogen, Carlsbad, CA, USA). Primary antibodies were diluted in blocking buffer unless otherwise noted. Secondary antibodies were donkey antisera to the primary antibody conjugated to Alexa Fluor (Life Technologies, Carlsbad, CA, USA) 488, 546 or 647. Antibody stained tissues were counterstained with DAPI (2-(4-amidinophenyl)-1H-indole-6-carboxamidine) or Hoechst 33342 to visualize nuclei. TUNEL assays were carried out on sections of exocrine tissue using the ApopTag In Situ detection kit (Chemicon, Temecula, CA, USA).

### Immunohistochemistry Quantification and Statistical Analysis

Quantitative analysis of protein expression on tissue sections was carried out by imaging 10 random fields of >200 nuclei per field spanning >100 microns of tissue depth for each treatment group. Fields were captured (Metamorph, Sunnyvale, CA, USA) and nuclei counting ImageJ (http://imagej.nih.gov) blinded to outcome. Significance was determined by Student’s T-test and reported as mean ± SEM (standard error of the mean) or ANOVA (analysis of variance) with Bonferroni-Holm post hoc analysis as indicated in the figure legends.

### Immunoprecipitation and Western Blotting

Cultured human adult exocrine tissue and E14.5 mouse fetal pancreatic epithelia were extracted in RIPA extraction buffer (0.05M Tris-HCl, pH 7.4, 0.15M NaCl, 0.25% deoxycholic acid, 1% NP-40, 1mM EDTA) supplemented with HALT protease and phosphatase inhibitor (Thermo Scientific, Waltham, MA, USA). Approximately 0.5 mg of human tissue protein lysate and lysate-free negative control were incubated overnight with 5 μg anti-mouse NGN3 F25A1B3 (concentrate) at 4°C. Bound proteins were isolated with magnetic beads covalently bound to Protein G (DYNAL, Carlsbad, CA, USA). Immunoprecipitated proteins and ~20μg E14.5 mouse pancreatic lysate were resolved on 12% HEPES/glycine/SDS gels and transferred to Immobilon-Fl PVDF membrane (Millipore, Billerica, MA, USA) and detected with F25A1B3 then reprobed with anti-E12/47. For coprecipitation of HES1 and ID proteins, approximately 0.5 mg of human tissue protein lysate was incubated overnight with 5 μg anti-HES1 (Cell Signaling, Beverly, MA, USA) at 4°C then immunoprecipitated and western blotted as above. Western blot strips were incubated in anti-ID1, ID2 and ID4 antibodies (Santa Cruz Biotechnology, Santa Cruz, CA, USA). Other antibodies used for protein detection were anti-V5 epitope (Sigma), anti-cleaved Notch 1 (Cell Signaling, Beverly, MA, USA) and anti-GAPDH (Millipore, Billerica, MA, USA).

### Recombinant Human NGN3 Expression

The coding region of human NGN3 cDNA was amplified by PCR from IMAGE Consortium clone 8992184 (NCBI accession BC126468) using an N-terminal primer which included the start codon and sequence to adapt the amplimer to the directional TOPO expression vector pENTER (Life Technologies, Waltham, MA, USA) (5’-CACCHGAAAGGATGACGCCTCAACCCTCGGG-3’). The C-terminal primer removed the NGN3 stop codon (5’-CAGAAAATCTGAGAAAGCCA-3’) allowing translation of V5 and 6X histidine tags. Gateway recombination was used to generate pHS.NGN3_V5HIS in the pDEST40 mammalian expression vector (Life Technologies, Carlsbad, CA, USA). DNA sequence verified pHS.NGN3_V5HIS was introduced into HEK293T cells using Lipofectamine 2000 (Life Technologies, Carlsbad, CA, USA). After 48 hours, recombinant protein was harvested by sonication in RIPA extraction buffer with protease and phosphatase inhibitors. Lysates were resolved on 12% polyacrylamide gels and western blotted.

### Electron Microscopy

Ultrathin sections of adult pancreas were stained with CD133 conjugated to magnetic beads (Miltenyi Biotec, Bergisch Gladbach, Germany) and imaged directly due to the iron content of the bead. CA19.9 was imaged with mouse anti-human CA19.9 (ab3982, IgM, abcam) and IgM specific gold beads.

### Preparation of Single Cells from Exocrine Tissue

Tissues were rinsed in calcium/magnesium-free PBS then dissociated by incubation in 0.05% trypsin/EDTA for 5 min. at 37°C with vigorous mixing. Following trypsin neutralization, DNA was digested by incubation at room temperature for ~2 min. with 120 units DNAse I per ml in media made 50 mM MgCl_2_ then passed over a 40 μm cell filter. Single cells were collected by centrifugation at 200xg for 5 min. then resuspended in 0.5% BSA, 2 mM EDTA prepared in calcium/magnesium-free PBS and counted by using a Nucleocounter (New Brunswick Scientific Enfield, CT, USA).

### Immunomagnetic CD133+ Cell Enrichment and Depletion

CD133 cell enrichment and depletion were carried out using antibody CD133/1 conjugated to magnetic beads (Miltenyi Biotec, Bergisch Gladbach, Germany) on SuperMACS and AutoMACS devices. Two rounds of magnetic bead enrichment were performed. CD133 enrichment and depletion levels were determined by staining with CD133/2 conjugated to phycoerythrin (CD133/2-PE) and FACS analysis with live gating using 7AAD. Total CD133+ cell number was determined in triplicate.

### FACS and Cytospin Analyses

To determine coexpression of NGN3 and CD133, single cell suspensions of exocrine tissue were divided in two aliquots. For cytospin analysis, one aliquot of cells was immunomagnetically enriched for CD133 then stained with mouse anti-CD133-PE at 4°C for 10 min., fixed in 2% PFA for 10 min. at room temperature and spun onto a slide using a cytospin. Slides were washed in 50 mM glycine/PBS, blocked with 5% donkey serum 1% BSA 0.1% triton X100, then stained with rabbit anti-human NGN3 or rabbit Ig (isotype negative control) diluted in blocking buffer overnight at 4°C. Proteins were visualized with donkey anti-rabbit Alexa Fluor 647 secondary antibody (Life Technologies, Carlsbad, CA, USA) and PE. Nuclei were stained with Hoechst 33342. For FACS analysis, the second aliquot of cells was stained with mouse anti-CD133-PE or IgG2b isotype negative control at 4°C for 10 min, then fixed, blocked and permeabilized using Transcription Factor Buffer Set (BD Pharmigen, San Jose, CA, USA) reagents. Cells were then stained with rabbit anti-human NGN3 or rabbit Ig then stained with anti-rabbit Alexa Fluor 647. Cell population was gated on FL2/SSC to identify CD133+ then analyzed for NGN3 expression in FL4.

### Transcriptome Analyses

Random-primed cDNA from CD133+ and CD133-depleted populations (n = 3) were subjected to >20 million DNA sequencing reads / per sample on an Illumina HiSeq2000 (San Diego, CA, USA) then analyzed using the TopHat-Cufflinks-Cuffdiff workflow [91]. Sequencing data was mapped with TopHat (v2.0.5) against the UCSC hg19 reference assembly. Mapping files were inputted into Cufflinks and Cuffdiff (V2.1.1) running on the Galaxy server (https://usegalaxy.org/) with geometric normalization, pooled dispersion and a false discovery rate (FDR) of 0.05 [91]. Differential expression results were ranked by fold change in fragments per kilobase of transcript per million mapped fragments (FPKM). Overall significance for each differential event detected (q) was based on *p* > FDR after Benjamini-Hochberg correction for multiple testing. Genes were reported as not expressed if the differential test failed due to not enough alignments, too high complexity or too shallow sequencing to carry out the test. Differential splicing, promoter usage and coding output are measured by the square root of the Jensen-Shannon divergence computed on the relative abundances of each overloading event. Global data analyses of the Cufflinks dataset were performed with CummRbund. To construct lists, all genes from transcriptional loci that could not be resolved to a single gene by Cuffdiff were added individually. Functional annotation of differentially expressed genes was carried out using the DAVID resource [92, 93]. Transcriptome data discussed in this publication have been deposited in NCBI's Gene Expression Omnibus and are accessible through GEO Series accession number GSE64854.

### Quantitative RTPCR

RNA from exocrine tissue and cells were prepared using the RNeasy miniprep kit (Qiagen, Venlo, Netherlands). cDNA was synthesized using oligo (dT) primers in a standard reverse transcriptase reaction and 5 μg RNA. Notch signaling pathway RTPCR profile was carried out using the RT^2^ Profiler PCR Array (Qiagen, PAHS-059YA, Venlo, Netherlands) using RNA from three exocrine cultures. All other mRNA levels were determined by using TaqMan (Applied Biosystems, Foster City, CA, USA) gene expression assays (S5 Table). Pancosphere cDNA was preamplified for 10 cycles using TaqMan PreAmp reagents (Applied Biosystems, Foster City, CA, USA) using a mixture of probe/primer sets to genes being detected. Mean levels (≥3 readings/sample) of genes of interest were normalized to mean levels of primer-attenuated cyclophillin A (PPIA). All human gene assays except ID2 were multiplexed with PPIA. Relative expression (RQ) was calculated using the ∆∆C_t_ method. Statistical analyses of expression were based on 2^- ∆Ct^ values from each treatment group. C_t_ values >35 cycles were treated as no expression. Mean relative expression levels for comparison of CD133+ and CD133D populations and CD133+ cell differentiation were carried out on RNA from four and three exocrine cultures, respectively.

### Pancosphere Culture and Maturation

To form pancospheres, CD133+ cells were resuspended at 2X10^5^ to 3X10^6^ cells/ml in base media [Knockout DMEM (Life Technologies, Carlsbad, CA, USA), 10% Knockout Serum Replacement (Life Technologies, Carlsbad, CA, USA), 10% human albumin, 10 ng/ml fibroblast growth factor 2, 1X Na pyruvate, and 1X non-essential amino acids] conditioned by overnight incubation on human SDEC cells [75, 94] then allowed to sit undisturbed in suspension culture for 4–6 days in low attachment plastic dishes. CD133+ cell plating was termed pancosphere day 0. Media was then changed by 30% volume change to maturation media [RPMI 1640, 0.5% BSA, 10 mM nicotinamide, 50 ng/ml human insulin like growth factor 2 (IGF2, R&D Systems, Minneapolis, MN, USA)] every three days until pancosphere day 19. IGF2 was then withdrawn with a 70–80% media change for 2 days then harvested on pancosphere day 21. When noted, maturation media was supplemented with 10 ∞M mycophenolic acid (TOCRIS Bioscience, Bristol, UK).

### Pancosphere Staining and Characterization

For identification and quantification cells within pancospheres, intact spheres were fixed and stained as whole mounts. All incubations and washes were carried out in buffers used to stain frozen sections and each step was carried out for ≥12 hours at 4°C. Stained pancospheres were mounted on glass slides and coverslipped. For quantitative analyses, unbiased stereology (Stereo Investigator Software, MBF Bioscience, Williston, VT, USA) was used to estimate number of nuclei per pancosphere. Pancospheres were imaged by confocal microscopy through the Z-axis using sequential acquisition mode. Immunoreactive cells were considered positive only if confocal orthogonal analysis demonstrated immunostaining that clearly surrounded a nucleus (cytoplasmic antigens) or if immunostaining colocalized cleanly within the nuclear counterstain (nuclear antigens). Mean PS diameter was calculated from 300 PS created from two CD133+ cell populations on PS day 6 and 20 from two CD133+ cell populations on PS day 15. Mean PS cell proliferation was measured by incorporation of nucleotide analogue EdU+ in 16 PS from two CD133+ cell populations. Mean Percentage of PS cells expressing NGN3+, PDX1 and CHGA was calculated in 12 PS from three CD133+ cell populations. The effect of mycophenolic acid on CPEP and GCG coexpression were investigated in 15 PS per treatment from three CD133+ preparations.

### Clonogenic Pancosphere Assay

CD133+ and CD133D cells from three CD133+ cell preparations were resuspended at 10 and 1000 cells per ml in SDEC conditioned media + 1% Matrigel (BD Biosciences, San Jose, USA) and 0.1 ml plated into each well of an ultra low attachment (Corning, Corning, NY, USA) 96 well plate. Wells containing one cell were highlighted for subsequent analysis. After 4 days, wells containing pancospheres were counted. PS formation was attempted from 100 CD133D cells/well from two cell preparations.

### Cytokine Array

Cytokine profile was carried out using a Ray Biotech (Norcross, GA, USA) G1000 human cytokine array of 120 cytokines according to manufacturers instructions. Three different batches of SDEC conditioned media were compared to base media.

### Ethics

Exocrine tissue and islets isolated from human pancreata of non-diabetic adult cadaver donors were obtained from ICR Basic Science Islet Distribution Program (IIDP) under the auspices of the National Institutes of Health National Center for Research Resources (NCRR). Written informed consent for research use was obtained by the institutions that collected the tissue. Tissues were received without personal identifiers. Based on the regulatory definition of human subject research, this study was granted and IRB exemption by the University of South Florida Institutional Review Board (FWA 00001669), review date 5/10/2011. Pancreas biopsies from living subjects undergoing medically indicated diagnostic procedures were obtained from Johns Hopkins Department of Pathology under IRB # NA_00001584, expiration date 8/11/2015.

## Supporting Information

S1 Fig Neurogenin 3 (NGN3) expression in human pancreas biopsies. (TIF)

S2 Fig Absence of CD133 expression in human islets. (TIF)

S3 Fig Full unedited gels. (TIF)

S1 Table Summary of pancreas biopsies from living patients and cadaveric organs. (DOCX)

S2 Table Differential transcript expression by CD133+ cells. (DOCX)

S3 Table Differential gene splicing, promoter usage and CDS output by CD133+ cells. (DOCX)

S4 Table Cytokine components of SDEC conditioned media. (DOCX)

S5 Table Quantitative PCR primer / probe sets. (DOCX)

S6 Table Abbreviations, Entrez Gene ID numbers and full names of target genes. (DOCX)
