# Supplementary figures and images for: Neurogenin 3 Expressing Cells in the Human Exocrine Pancreas Have the Capacity for Endocrine Cell Fate
Source: PLoS One. 2015 Aug 19;10(8):e0133862. doi: 10.1371/journal.pone.0133862 (PMC4545947; doi:10.1371/journal.pone.0133862)

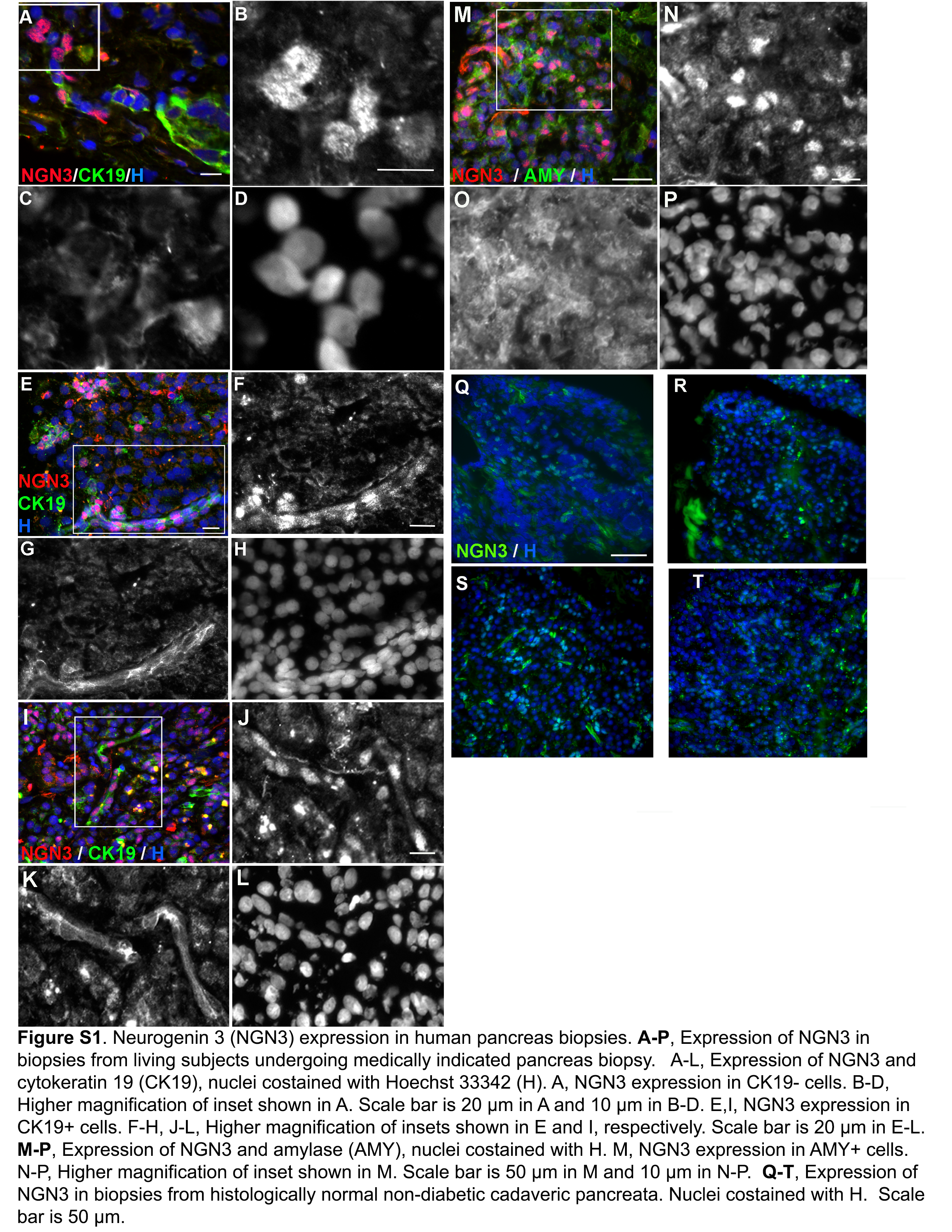

Supplement: S1 Fig — (TIF) [file pone.0133862.s001.tif]

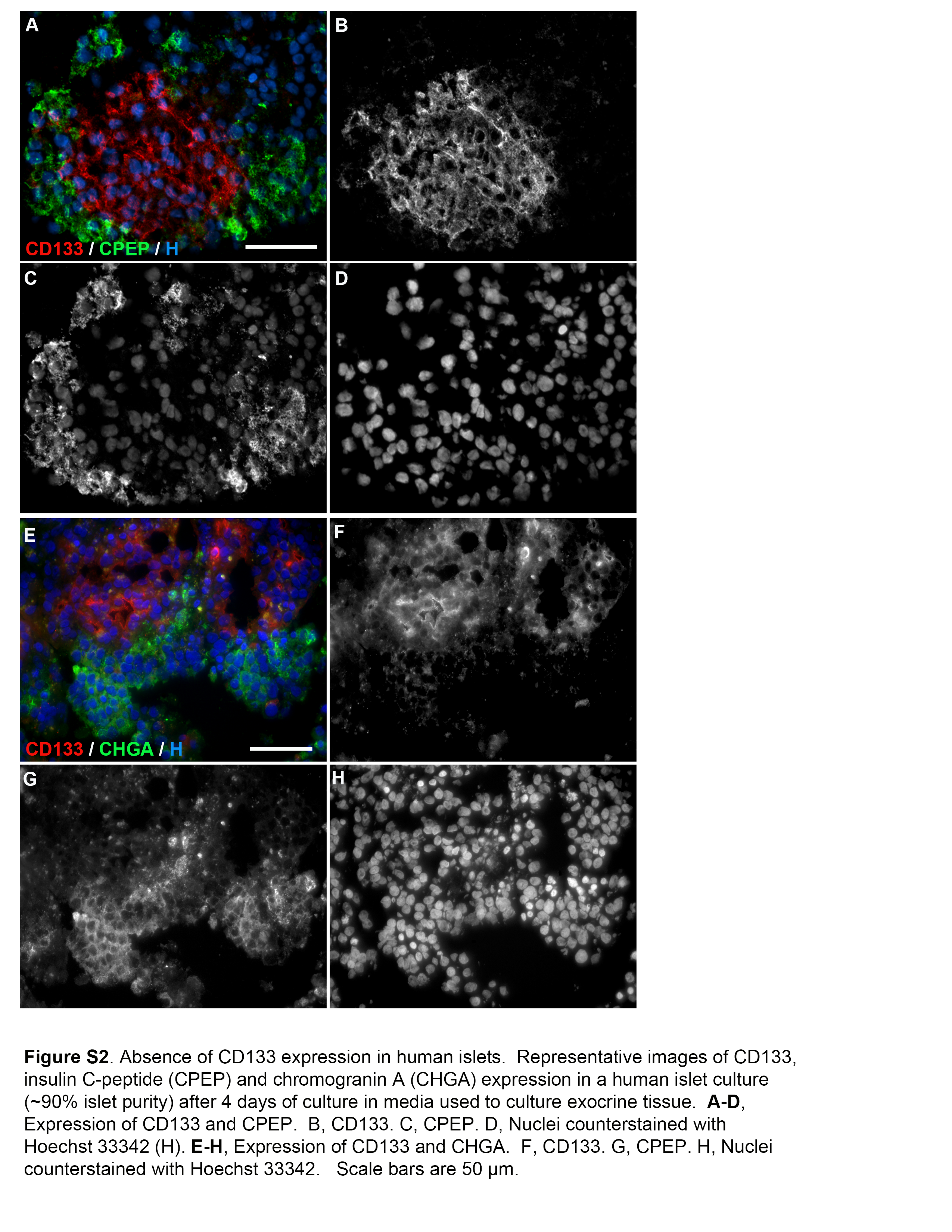

Supplement: S2 Fig — (TIF) [file pone.0133862.s002.tif]

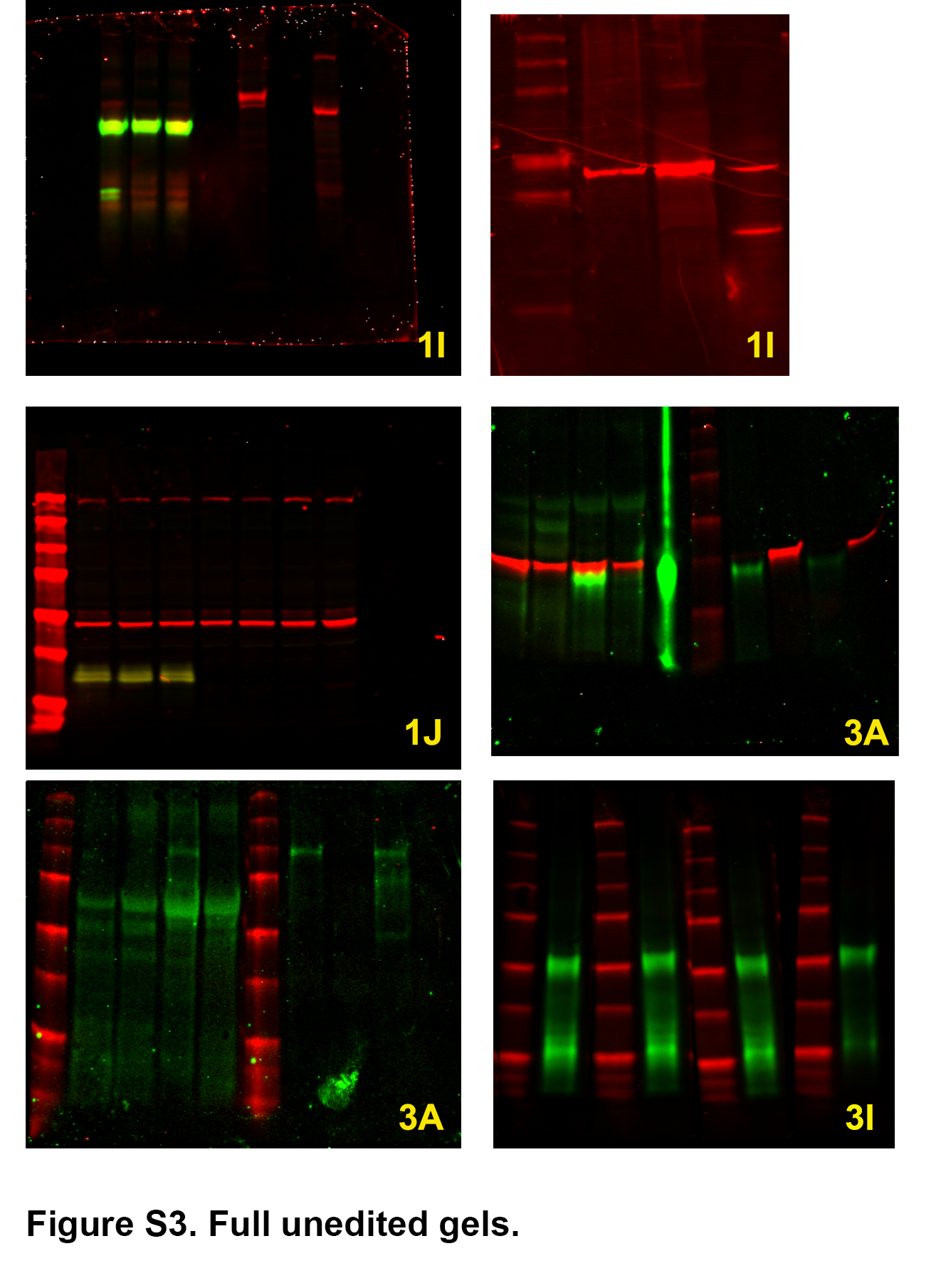

Supplement: S3 Fig — (TIF) [file pone.0133862.s003.tif]
