# Supplementary material for: Neurogenin 3 Expressing Cells in the Human Exocrine Pancreas Have the Capacity for Endocrine Cell Fate
Source: PLoS One. 2015 Aug 19;10(8):e0133862. doi: 10.1371/journal.pone.0133862 (PMC4545947; doi:10.1371/journal.pone.0133862)
Supplement: S1 Table — (DOCX) [file pone.0133862.s004.docx]

**S1 .** Summary of pancreas biopsies from living patients and cadaveric organs

|  |  |  |  | % NGN3 | |
| --- | --- | --- | --- | --- | --- |
|  |  |  |  | F25A1B3 | HPA039785 |
| Type | ID | Time to biopsy (min) | Age (yr) | Mean ± SE | Mean ± SE |
| Living | 747 | ND | 77 | 1.0 ± 0.1 | ND |
| Living | 839 | ND | 73 | 2.2 ± 0.1 | ND |
| Living | 421 | ND | 65 | 0.3 ± 0.0 | ND |
| Living | 271 | ND | 65 | 6.5 ± 0.6 | ND |
| Living | 159 | ND | 27 | 2.2 ± 0.2 | ND |
| Cadaver | H460 | 339 | 48 | 11.1 ± 1.1 | 12 ± 0.9 |
| Cadaver | H461 | 677 | 18 | 11.6 ± 1.4 | 11.0 ± 1.0 |
| Cadaver | H462 | 230 | 54 | 9.5 ± 0.6 | 9.6 ± 0.6 |
| Cadaver | H465 | 483 | 47 | 8.6 ± 0.8 | 11.7 ± 1.3 |

Living biopsies were from histologically normal regions of pancreata with some underlying pathology except case 159, which was taken due to splenic invagination. Type, Biopsy from living patient or cadaver. ID, Sample reference ID, Time to biopsy, Time in minutes from organ removal to biopsy. Age, Age in years of patient. %NGN3, Mean ± SEM (n=10 fields/biopsy) percentage of NGN3+ nuclei in each biopsy stained with anti-mouse NGN3 (F25A1B3) and anti-human NGN3 (HPA039785) antibodies. ND, Not determined.
