# Supplementary material for: Neurogenin 3 Expressing Cells in the Human Exocrine Pancreas Have the Capacity for Endocrine Cell Fate
Source: PLoS One. 2015 Aug 19;10(8):e0133862. doi: 10.1371/journal.pone.0133862 (PMC4545947; doi:10.1371/journal.pone.0133862)
Supplement: S2 Table — (DOCX) [file pone.0133862.s005.docx]

**S2 Table.** **Differential transcript expression by CD133+ cells.**

|  | Gene | | | | Isoform | | | |  | CDS | | | | TSS | | | |
| --- | --- | --- | --- | --- | --- | --- | --- | --- | --- | --- | --- | --- | --- | --- | --- | --- | --- |
|  | 1727 (87 unique) | | |  | 1194 (0 unique) | | | |  | 1268 (0 unique) | | | | 1530 (4 unique) | | | |
|  | Up | | Down | | Up | | Down | | | Up | | Down | | Up | | Down | |
| CD133+/CD133D | Gene | Fold | Gene | Fold | Gene | Fold | Gene | Fold | | Gene | Fold | Gene | Fold | Gene | Fold | Gene | Fold |
|  | **VTCN1** | 881.7 | **PPY** | 573.2 | VTCN1 | 881.7 | PPY | 573.2 | | VTCN1 | 881.7 | PPY | 573.2 | PROM1 | 1357.9 | DCN | 134859.7 |
|  | **CFTR** | 371.1 | **GCG** | 408.2 | CFTR | 371.1 | GCG | 408.2 | | CFTR | 371.1 | GCG | 408.2 | VTCN1 | 881.7 | **INS/IGF2** | 1947.8 |
|  | **MUC15** | 313.5 | **TTR** | 301.6 | HSD17B2 | 194.9 | TTR | 301.6 | | HSD17B2 | 194.9 | TTR | 301.6 | CFTR | 371.1 | PPY | 573.2 |
|  | **NTRK2** | 277.1 | **COL6A3** | 155.7 | VAV3 | 187.6 | COL6A3 | 169.7 | | VAV3 | 187.6 | COL6A3 | 169.7 | MUC15 | 313.5 | GCG | 408.2 |
|  | **HSD17B2** | 194.9 | **GPNMB** | 153.4 | UPK1B | 155.3 | GPNMB | 125.1 | | BARX2 | 159.1 | GPNMB | 125.1 | HSD17B2 | 194.9 | TTR | 301.6 |
|  | **PROM1** | 191.1 | **DCN** | 122.9 | AQP1 | 145.1 | COL1A2 | 119.5 | | UPK1B | 155.3 | COL1A2 | 119.5 | VAV3 | 187.6 | COL6A3 | 155.7 |
|  | **VAV3** | 187.6 | **COL1A2** | 119.5 | TINAGL1 | 133.2 | FBN1 | 100.7 | | PPARGC1A | 145.9 | FBN1 | 100.7 | BARX2 | 159.1 | GPNMB | 153.4 |
|  | **BARX2** | 159.1 | **FBN1** | 100.7 | MMP7 | 117.3 | THBS2 | 96.9 | | AQP1 | 145.1 | THBS2 | 96.9 | UPK1B | 155.3 | COL1A2 | 119.5 |
|  | **AQP1** | 157.0 | **THBS2** | 96.9 | SERPINA5 | 98.9 | COL3A1 | 93.4 | | TINAGL1 | 133.2 | COL3A1 | 93.4 | PPARGC1A | 145.9 | FBN1 | 100.7 |
|  | **UPK1B** | 155.3 | **COL3A1** | 93.4 | SERPINA6 | 92.3 | BGN | 93.4 | | MMP7 | 117.3 | BGN | 93.4 | AQP1 | 145.1 | THBS2 | 96.9 |
|  | **PPARGC1A** | 145.9 | **BGN** | 93.4 | KRT23 | 88.3 | SFRP2 | 90.5 | | SERPINA5 | 98.9 | SFRP2 | 90.5 | TINAGL1 | 133.2 | **BCAT1** | 94.6 |
|  | **TINAGL1** | 136.2 | **SFRP2** | 90.5 | NPPB | 85.7 | FAP | 88.9 | | SERPINA6 | 92.3 | FN1 | 89.9 | VCAM1 | 127.8 | COL3A1 | 93.4 |
|  | **VCAM1** | 127.8 | **HSD11B1** | 89.4 | **SERPINA1** | 82.9 | COL5A2 | 86.4 | | KRT23 | 88.3 | HSD11B1 | 89.4 | MMP7 | 117.3 | BGN | 93.4 |
|  | **MMP7** | 117.3 | **FAP** | 88.9 | S100A14 | 82.0 | LUM | 86.0 | | NPPB | 85.7 | FAP | 88.9 | SERPINA5 | 98.9 | SFRP2 | 90.5 |
|  | **SERPINA5** | 98.9 | **COL5A2** | 86.4 | **C15orf52** | 75.9 | COL15A1 | 81.5 | | S100A14 | 82.0 | COL5A2 | 86.4 | SERPINA6 | 92.3 | FAP | 88.9 |
|  | **SERPINA6** | 92.3 | **LUM** | 86.0 | **ANXA3** | 72.0 | PDGFRB | 76.6 | | SERPINA1 | 77.3 | LUM | 86.0 | KRT23 | 88.3 | COL5A2 | 86.4 |
|  | **KRT23** | 88.3 | **FN1** | 84.8 | **NMB** | 64.6 | **SPOCK1** | 75.4 | | C15orf52 | 75.9 | COL15A1 | 81.5 | NPPB | 85.7 | LUM | 86.0 |
|  | **NPPB** | 85.7 | **COL15A1** | 81.5 | **SCNN1A** | 60.3 | **IGFBP5** | 75.1 | | ANXA3 | 72.0 | PDGFRB | 76.6 | SERPINA1 | 82.9 | FN1 | 84.8 |
|  | **S100A14** | 82.0 | **VCAN** | 77.1 | **ANXA9** | 57.4 | **A2M** | 63.9 | | NMB | 64.6 | SPOCK1 | 75.4 | S100A14 | 82.0 | COL15A1 | 81.5 |
|  | **FXYD2/6** | 81.6 | **PDGFRB** | 76.6 | **DAPL1** | 54.5 | **COL1A1** | 63.3 | | SCNN1A | 60.3 | **IGFBP5** | 75.1 | C15orf52 | 75.9 | **VCAN** | 77.1 |

Bold indicates initial listing in this table.

Transcriptome analysis of 20 highest differentially expressed gene, isoform, coding sequence (CDS) and transcriptional start site (TSS) differences in CD133+ cells compared to CD133-depleted cells (CD133D) isolated after four days of exocrine tissue culture. Up, Increased expression level in CD133+ cells compared to CD133D. Down, Decreased expression level in CD133+ cells compared to CD133D. Total number of significant (q<0.05, n=3 exocrine cultures) transcripts in each category and number of transcripts unique to each category are indicate. Significance (q) was calculated after Benjamini-Hochberg correction for multiple testing. Fold, Fold difference in expression of each gene between CD133+ and CD133D populations.
