# Supplementary material for: Neurogenin 3 Expressing Cells in the Human Exocrine Pancreas Have the Capacity for Endocrine Cell Fate
Source: PLoS One. 2015 Aug 19;10(8):e0133862. doi: 10.1371/journal.pone.0133862 (PMC4545947; doi:10.1371/journal.pone.0133862)
Supplement: S3 Table — (DOCX) [file pone.0133862.s006.docx]

**Table S6.** **Cytokine components of SDEC conditioned media**

| Cytokine | Fold | Cytokine | Fold |
| --- | --- | --- | --- |
| GM-CSF | 2501.6 | FGF-7 | 11.7 |
| IL-6 | 1360.2 | GCSF | 11.4 |
| GCP-2 | 1086.8 | MIP-1alpha | 8.6 |
| MCP-3 | 572.3 | PIGF | 8.6 |
| Osteoprotegerin | 490.0 | Angiopoietin-2 | 7.0 |
| IL-1beta | 463.2 | ICAM-3 | 6.7 |
| Angiogenin | 351.1 | TECK | 6.1 |
| HGF | 349.6 | M-CSF | 6.1 |
| MCP-1 | 294.5 | TGF-beta 3 | 5.9 |
| MIP-3-alpha | 255.5 | sgp130 | 5.8 |
| MCP-2 | 234.1 | Leptin | 5.7 |
| RANTES | 220.7 | MIF | 5.2 |
| GRO | 208.7 | CCL-28 | 4.6 |
| GRO-alpha | 208.3 | MIP-1-delta | 4.1 |
| MMP-1 | 199.6 | MCP-4 | 4.0 |
| IGFBP-2 | 165.8 | BTC | 3.4 |
| TIMP-2 | 158.8 | Endoglin | 3.3 |
| Activin A | 98.6 | IL-10 | 3.2 |
| IGFBP-4 | 90.4 | TIMP-1 | 3.1 |
| LAP | 81.3 | IGFBP-3 | 3.1 |
| IL-8 | 63.0 | sTNF-RI | 3.0 |
| IGFBP-1 | 43.8 | Axl | 3.0 |
| ENA-78 | 42.6 | Amphiregulin | 2.9 |
| VEGF | 39.5 | TIMP-4 | 2.9 |
| EGF | 37.3 | IGF-I SR | 2.7 |
| I-309 | 30.5 | Thrombopoietin | 2.7 |
| IL-1alpha | 23.1 | GITR-Ligand | 2.7 |
| uPAR | 22.2 | GDNF | 2.6 |
| PDGF AA | 20.2 | VEGF-D | 2.6 |
| IL-2 Ralpha | 18.7 | Lymphotactin | 2.5 |
| IGFBP-6 | 16.9 | ICAM-2 | 2.5 |
| Eotaxin | 15.1 | TRAIL R3 | 2.4 |
| PDGF-BB | 14.0 | SCF | 2.1 |
| IL-11 | 13.4 | IL-7 | 2.1 |
| LIF | 12.8 |  |  |

Cytokine levels determined by antibody array. Fold, Mean (n=3 SDEC cell cultures) fold difference in concentration of each cytokine in media conditioned by SDEC cells compared to unconditioned base media. Cytokines <2-fold higher than base media not shown.
