# Supplementary material for: Neurogenin 3 Expressing Cells in the Human Exocrine Pancreas Have the Capacity for Endocrine Cell Fate
Source: PLoS One. 2015 Aug 19;10(8):e0133862. doi: 10.1371/journal.pone.0133862 (PMC4545947; doi:10.1371/journal.pone.0133862)
Supplement: S5 Table — (DOCX) [file pone.0133862.s008.docx]

**S5 Table.** **Quantitative PCR primer / probe sets**

| Gene | Item |
| --- | --- |
| CHGA | Hs00900375_m1 |
| PPIA | 4310883E |
| FOXA2 | Hs00232764_m1 |
| FOXO1 | Hs01054576_m1 |
| GATA4 | Hs00171403_m1 |
| GLIS3 | Hs00541450_m1 |
| HES1 | Hs00172878_m1 |
| HHEX | Hs00242160_m1 |
| HNF1B | Hs01001602_m1 |
| INS | Hs02741908_m1 |
| ISL1 | Hs00158126_m1 |
| KI67 | Hs01032443_m1 |
| MAFA | Hs01651425_s1 |
| MAFB | Hs00534343_s1 |
| MNX1 | Hs00907365_m1 |
| NEUROD1 | Hs01922995_s1 |
| NGN3 | Hs01875204_s1 |
| NKX6.1 | Hs00232355_m1 |
| NKX2.2 | Hs00159616_m1 |
| ONECUT1 | Hs00413554_m1 |
| ONECUT2 | Hs00191477_m1 |
| PAX4 | Hs00173014_m1 |
| PAX6 | Hs00240871_m1 |
| PDX1 | Hs00236830_m1 |
| PTF1A | Hs00603586_g1 |
| SOX9 | Hs01001343_g1 |
