# Supplementary material for: Neurogenin 3 Expressing Cells in the Human Exocrine Pancreas Have the Capacity for Endocrine Cell Fate
Source: PLoS One. 2015 Aug 19;10(8):e0133862. doi: 10.1371/journal.pone.0133862 (PMC4545947; doi:10.1371/journal.pone.0133862)
Supplement: S6 Table — (DOCX) [file pone.0133862.s009.docx]

**.** **Abbreviations, Entrez Gene ID numbers and full names of target genes**

| Gene | GENE ID | Official full name (common or other name) |
| --- | --- | --- |
| BDNF | 627 | Brain-derived neurotrophic factor |
| CDH1 | 999 | Cadherin 1, type 1 (E-cadherin) |
| CFTR | 1080 | Cystic fibrosis transmembrane conductance regulator |
| CHGA | 1113 | Chromogranin A |
| CPEP | 3630 | Insulin C-peptide |
| DLL1 | 28514 | Delta-like 1 |
| DLL3 | 10683 | Delta-like 3 |
| DLL4 | 54567 | Delta-like 4 |
| E12/47 | 6929 | Transcription factor 3 (TCF3) |
| FBW7 | 50754 | F-box and WD-40 domain protein 7 (FBXW7) |
| FOXA2 | 3170 | Forkhead box A2 |
| FOXO1 | 2308 | Forkhead box O1 |
| GATA4 | 2626 | GATA binding protein 4 |
| GAPDH | 2597 | Glyceraldehyde 3-phosphate dehydrogenase |
| GCG | 2641 | Glucagon (GCG) |
| GLIS3 | 169792 | GLIS family zinc finger 3 |
| HES1 | 3280 | Hes family bHLH transcription factor 1 |
| HHEX | 3087 | Hematopoietically expressed homeobox |
| HNF1B | 6928 | HNF1 homeobox B |
| ID1 | 3397 | Inhibitor of DNA binding 1 |
| ID2 | 3398 | Inhibitor of DNA binding 2 |
| ID3 | 3399 | Inhibitor of DNA binding 3 |
| ID4 | 3400 | Inhibitor of DNA binding 4 |
| INS | 3630 | Insulin |
| ISL1 | 3670 | ISL LIM homeobox 1 |
| JAG1 | 182 | Jagged 1 |
| JAG2 | 3714 | Jagged 2 |
| KI67 | 4288 | Marker of proliferation Ki-67 |
| MAFA | 389692 | v-maf avian musculoaponeurotic fibrosarcoma oncogene A |
| MAFB | 9935 | v-maf avian musculoaponeurotic fibrosarcoma oncogene B |
| MMP7 | 4316 | Matrix metallopeptidase 7 (matrilysin) |
| MNX1 | 3110 | Motor neuron and pancreas homeobox 1 (HB9) |
| NEUROD1 | 4760 | Neuronal differentiation 1 |
| NGN3 | 50674 | Neurogenin 3 (NEUROG3) |
| NKX6.1 | 4825 | NK6 homeobox 1 |
| NKX2.2 | 4821 | NK2 homeobox 2 |
| Notch1 | 4851 | Notch 1 |
| Notch2 | 4853 | Notch 2 |
| Notch3 | 4854 | Notch 3 |
| Notch4 | 4855 | Notch 4 |
| ONECUT1 | 3175 | Onecut homeobox 1 (HNF6α) |
| ONECUT2 | 9480 | Onecut homeobox 2 |
| PAX4 | 5078 | Paired box 4 |
| PAX6 | 5080 | Paired box 6 |
| PDX1 | 3651 | Pancreatic and duodenal homeobox 1 (IPF1) |
| PPIA | 5478 | Peptidylprolyl isomerase A (cyclophillin A) |
| PRSS1 | 5644 | Protease, serine 1 (trypsin 1) |
| PRSS2 | 5645 | Protease, serine 1 (trypsin 2) |
| PTF1A | 256297 | Pancreas specific transcription factor 1A |
| PROM1 | 8842 | Prominin 1 (CD133) |
| REG1A | 5967 | Regenerating islet-derived 1 alpha |
| REG1B | 5968 | Regenerating islet-derived 1 beta |
| SERPINA3 | 12 | Serpin peptidase inhibitor clade A (alpha-1 antiproteinase, antitrypsin), member 3 |
| SOX9 | 6662 | SRY (sex determining region Y)-box 9 |
| SPINK1 | 6690 | Serine peptidase inhibitor Kazal type 1(pancreatic secretory trypsin inhibitor 1) |
|  |  |  |
